# In Vitro Effects of *Streptococcus oralis* Biofilm on Peri-Implant Soft Tissue Cells

**DOI:** 10.3390/cells9051226

**Published:** 2020-05-15

**Authors:** Alexandra Ingendoh-Tsakmakidis, Jörg Eberhard, Christine S. Falk, Meike Stiesch, Andreas Winkel

**Affiliations:** 1 Department of Prosthetic Dentistry and Biomedical Materials Science, Hannover Medical School, Carl-Neuberg-Str. 1, 30625 Hannover, Germany; joerg.eberhard@sydney.edu.au (J.E.); stiesch.meike@mh-hannover.de (M.S.); winkel.andreas@mh-hannover.de (A.W.); 2 Institute of Transplant Immunology, Hannover Medical School, Carl-Neuberg-Str. 1, 30625 Hannover, Germany; falk.christine@mh-hannover.de

**Keywords:** *Streptococcus oralis*, commensal, co-culture, biofilm, peri-implant mucosa, human gingival epithelial cells, human gingival fibroblasts

## Abstract

Human gingival epithelial cells (HGEps) and fibroblasts (HGFs) are the main cell types in peri-implant soft tissue. HGEps are constantly exposed to bacteria, but HGFs are protected by connective tissue as long as the mucosa–implant seal is intact. *Streptococcus oralis* is one of the commensal bacteria, is highly abundant at healthy implant sites, and might modulate soft tissue cells—as has been described for other streptococci. We have therefore investigated the effects of the *S. oralis* biofilm on HGEps and HGFs. HGEps or HGFs were grown separately on titanium disks and responded to challenge with *S. oralis* biofilm. HGFs were severely damaged after 4 h, exhibiting transcriptional inflammatory and stress responses. In contrast, challenge with *S. oralis* only induced a mild transcriptional inflammatory response in HGEps, without cellular damage. HGFs were more susceptible to the *S. oralis* biofilm than HGEps. The pro-inflammatory interleukin 6 (IL-6) was attenuated in HGFs, as was interleukin 8 (CXCL8) in HGEps. This indicates that *S. oralis* can actively protect tissue. In conclusion, commensal biofilms can promote homeostatic tissue protection, but only if the implant–mucosa interface is intact and HGFs are not directly exposed.

## 1. Introduction

The insertion of dental implants to restore missing teeth is a widespread practice [1] and titanium is currently the material of choice [2]. ‘Healthy’ implants possess a well-developed soft tissue seal [3,4,5,6]. This soft tissue consists of an epithelial layer at the tissue border, as well as underlying connective tissue, mainly including gingival fibroblasts and collagen [7]. The epithelial cells actively participate in re-epithelialization, and in innate and antimicrobial host defense [8]. Gingival fibroblasts exert a similar role, and are responsible for the turnover of connective tissue and the inflammatory response [9,10,11]. Many studies on infection have employed epithelial cells, which cover the whole soft tissue [12,13,14,15,16,17]. However, after injury, during the initial stage of implant insertion or in peri-implant disease, gingival fibroblasts too may come into contact with oral bacteria [10], which attach to implant materials and form biofilms [18]. Subsequently, the healthy peri-implant mucosa builds an essential barrier against biofilm penetration [19].

Dental implants are colonized by oral bacteria, mainly streptococci, as soon as 30 min after insertion [18]. In healthy implants, biofilms are primarily composed of commensal bacteria species, such as *Streptococcus oralis*, the predominant initial colonizer [3,4,5,6,20,21,22,23,24,25]. Previous studies have addressed the roles of these bacteria, besides their participation in initial colonization [25,26,27,28,29,30,31,32,33,34,35,36,37,38,39,40]. For instance, the role of *S. oralis* in systemic diseases—including bacteremia and cardiovascular diseases—has been extensively studied [25,26,27,28,29,30,31,32]. Moreover, *S. oralis* serves as an anchor for intermediate and late pathogenic colonizers [33,34], and can enhance bacterial pathogenicity [36] and the virulence of *Candida albicans* [21,36,37,38]. Nevertheless, it has been shown that *S. oralis* can antagonize bacterial pathogens and thereby promote homeostasis [39,40]. Although previous studies have shown that other streptococci may modulate the host and thus lead to tissue protection [16,41,42,43,44,45,46], little is known about how an *S. oralis* biofilm could modulate peri-implant tissue. As *S. oralis* is highly abundant at implant sites and human gingival fibroblasts (HGFs) and human gingival epithelial cells (HGEps) are the main cell types in peri-implant tissue, we have investigated the effects of *S. oralis* biofilm on these cell types.

We hypothesized that *S. oralis* biofilm has modulatory effects on peri-implant soft tissue cells—as observed for other streptococci. We assumed that host modulation by *S. oralis* would similarly promote tissue protection against destruction to that demonstrated for the other *Streptococcus* spp. Moreover, we expected that HGFs and HGEps would exhibit distinct responses to the commensal *S. oralis* biofilm, in accordance with their tissue topography. As already described, HGEps are located at tissue borders and, during evolution, they were in constant contact with bacteria and other factors in the oral cavity [16,46]. In contrast, HGFs reside in connective tissue—as long as tissue integrity is preserved [7,10]. We therefore compared the responses of HGFs and HGEps.

## 2. Materials and Methods

### 2.1. Formation of Streptococcus oralis Biofilm

*Streptococcus oralis* DSM 20627 (DSMZ, Braunschweig, Germany) was grown at 37 °C in tryptic soy broth (TSB, CM0129, Oxoid, Hampshire, UK), supplemented with 0.3% *w/v* yeast extract (Carl Roth GmbH + Co. KG, Karlsruhe, Germany), for 18 hours under anaerobic conditions (80% N_2_, 10% H_2_, 10% CO_2_, Don Whitley Scientific Limited, Shipley, UK) and with stirring. The *S. oralis* biofilm was grown on supporting hydrophilic polyethersulfone membrane (Merck Millipore, Darmstadt, Germany). Sterile membranes with 13 mm diameter were placed into 24-well plates with the dull side up and washed in 1 mL PBS for 18 hours under anaerobic conditions with parallel shaking. The overnight bacterial culture was centrifuged at 4,000× *g* for 15 min at 4 °C and diluted in brain heart infusion broth (BHI, CM1135, Oxoid, Hampshire, UK), supplemented with 5% *w/v* D(+)-saccharose (4621.2, Carl Roth GmbH, Karlsruhe, Germany) to give an optical density of 0.06 at 600 nm, corresponding to 8.7 × 10^7^CFU/mL. This *S. oralis* suspension was added on top of the membranes (600 μL/well). For the control groups, the sterile culture medium was used. Membranes were incubated at 37 °C in a humidified atmosphere with 5% CO_2_ for 72 hours. The medium was replenished on alternate days.

### 2.2. Human Tissue Cell Culture and Titanium Colonization

Primary human gingival fibroblasts (HGF) were purchased from Provitro AG (1210412, Berlin, Germany). The HGFs were cultured in Dulbecco’s Modified Eagle Medium (DMEM, FG0435, Merck Millipore, Darmstadt, Germany) supplemented with 10% FBS and 1% *v/v* penicillin/streptomycin at 37 °C in a humidified atmosphere with 5% CO_2_. The cells were passaged after reaching 80–90% confluency. Primary human gingival epithelial cells (HGEps) were purchased from CELLnTEC Advanced Cell Systems AG (HGEPp-05, Bern, Switzerland). The HGEps were cultured in CnT Prime medium (CnT-PR, CELLnTEC Advanced Cell Systems AG, Bern, Switzerland) at 37 °C in a humidified atmosphere with 5% CO_2_. The cells were passaged at 70–80% confluency.

To simulate the adherence of mucosal tissue to the titanium implant, we colonized titanium disks with the main cell types, HGF or HGEp. The two cell types were grown separately on titanium disks (grade 4, Ø 12 mm, ground with 45 μm profile). Sterile titanium disks were placed into 24-well plates and washed once with DMEM or CnT Prime. The HGFs were seeded onto the titanium disks at a density of 15,000 cells per mL per well in fully supplemented DMEM. The HGEps were seeded at a density of 45,000 cells per mL per well in CnT Prime medium. Titanium disks were incubated at 37 °C in a humidified atmosphere with 5% CO_2_ for four days with a single medium replacement after two days.

### 2.3. Co-Culture of Tissue Cells Grown on Titanium with Streptococcus oralis Biofilm

**Figure 1:**
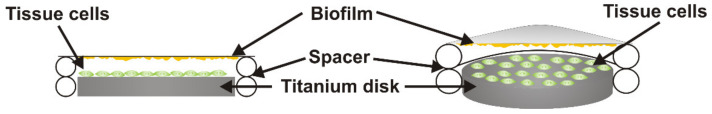
Schematic representation of the co-culture set up. Titanium disks were colonized by primary human gingival epithelial cells (HGEps) or fibroblasts (HGFs) (green). The *S. oralis* biofilm (yellow) was placed on top of spacers and was facing the tissue cell monolayers at a distance of 1 mm. The well was filled with an antibiotic-free cell culture medium. Left: 2D cross-section of the co-culture set up. Right: 3D representation with a cross-section of spacer and biofilm.

The cell-colonized titanium disks were transferred into 24-well plates with 1 mL fresh culture medium without antibiotics. The DMEM medium without any additions was used for HGFs and the CnT prime medium for HGEps. Sterile spacers were placed around the titanium disks. The *S. oralis* biofilms and the control membranes were washed five times with sterile PBS and then placed on the spacers facing the cells grown on the titanium disks (Figure 1). The distance between biofilm and tissue cells was 1 mm. These co-culture constructs were incubated at 37 °C in a humidified atmosphere with 5% CO_2_ for a period of 2, 4, or 6 h. At each time point, samples were processed for analysis.

### 2.4. Qualitative LIVE/DEAD Staining and Quantitative Analysis of Cell Attachment of Gingival Tissue Cells after Biofilm Challenge

A LIVE/DEAD staining of tissue cells was performed after co-culture with the *S. oralis* biofilm. For the staining solution, calcein AM (C3099, Thermo Fisher Scientific Inc., Waltham, USA) and propidium iodide (P4864, Sigma-Aldrich Corporation, St. Louis, USA) were diluted 1:1000 in sterile PBS. The co-culture supernatants were carefully collected, and 1 mL of the LIVE/DEAD staining solution was added for 30 min at 37 °C in a humidified atmosphere with 5% CO_2_. The staining solution was removed and replaced by PBS for microscopic examination using CLSM (Confocal Scanning Laser Microscope, Leica TCS SP2 or Leica TCS SP8, Leica Microsystems, Wetzlar, Germany).

After examination of the LIVE/DEAD staining, the tissue cells were fixed with 4% *v/v* paraformaldehyde (335.2, Carl Roth GmbH, Karlsruhe, Germany) in PBS for 10 min. The samples were carefully washed with PBS and permeabilized with 1% *v/v* triton-X-100 (93418, Sigma-Aldrich Corporation, St. Louis, USA) in PBS for 10 min followed by a wash with PBS. Then, the cell nuclei were stained with 1 µg/mL 4′,6-Diamidino-2-phenylindole dihydrochloride (DAPI, D9542, Sigma-Aldrich Corporation, St. Louis, USA) in PBS for 15 min. All steps were performed at room temperature and in the dark. The stained samples were washed with PBS and five random images were acquired using CLSM (Confocal Scanning Laser Microscope, Leica TCS SP8, Leica Microsystems, Wetzlar, Germany). All images of cell nuclei were automatically counted using ImageJ (http://rsbweb.nih.gov/ij/), a public domain Java image processing program [47]. Briefly, images were converted to an 8-bit format, the threshold was adjusted to exclude background and then the smooth tool was applied. The watershed tool was applied to separate closely attached cell nuclei before automatic particle analysis. Resulting cell numbers were used for further statistical analysis. The assumption that one cell nucleus corresponds to one cell was made.

### 2.5. RNA Extraction of Tissue Cells

The RNeasy ^®^ Mini Kit from QIAGEN (74104, Hilden, Germany) was used for RNA isolation from the gingival tissue cells. The isolation was performed with the DNase I treatment according to the manufacturer’s instructions. The RLT buffer was supplemented with 1% (*v/v*) β-mercaptoethanol (M6250, Sigma-Aldrich Corporation, St. Louis, USA) and tissue cells were scratched with a cell scraper from the disk surface.

### 2.6. Array Hybridization and Data Analysis

The hybridization was performed on the Whole Genome Oligo Microarray Kit 4x44K v2 including 44495 probes with roughly 26,000 transcripts. The aminoallyl-UTP-modified (aaUTP) cRNA was prepared using 70 ng total RNA and halved reaction volumes according to the manufacturer’s protocol (AM1753, Life Technologies, Carlsbad, USA). The labeling of the aaUTP-cRNA was performed using the Alexa Fluor 555 Reactive Dye. Before the reverse transcription reaction, 1 µL of Agilent’s One-Color spike-in Kit stock solution (1:100,000) was added to each single RNA sample. For the hybridization, 500 ng of labeled cRNA was used. The cRNA fragmentation, hybridization, and washing steps were followed using the One-Color Microarray-Based Gene Expression Analysis Protocol V5.7. Slide scanning was performed on the Agilent Micro Array Scanner G2565CA (Agilent Scientific Instruments, Santa Clara, USA) pixel resolution 5 µm, bit depth 20) and the data were extracted with the Feature Extraction Software V10.7.3.1 (extraction protocol file GE1_107_Sep09.xlm). The microarray-based data are available in Gene Expression Omnibus (GEO); the related accession number is GSE134481.

The gene expression data were analyzed using the Qlucore Omics Explorer (Qlucore AB, Lund, Sweden) bioinformatics software. The two-group comparison (t-test) was applied to filter the gene expression data of the control and biofilm treated groups. Before analysis, the replicate factor was eliminated; the *p*-value was set to 0.01, and the fold change filter was set to 2.

The pathway analysis was performed using DAVID (Database for Annotation, Visualization, and Integrated Discovery) [48,49]. The generated gene lists after analysis with the Qlucore Omics Explorer were submitted to DAVID. The lists of up- and down-regulated genes after *S. oralis* biofilm challenge were uploaded separately and pathway analysis was performed with default settings. Differentially regulated genes within the enriched pathway were designated to their role in the pathway according to the KEGG (Kyoto Encyclopedia of Genes and Genomes) [50,51,52] and were visualized with Pathview, a tool for pathway-based data integration and visualization [53,54].

### 2.7. Cytokine Detection in Co-Culture Supernatants

To quantify the cytokines in the co-culture supernatants, they were centrifuged at 4,000× *g* for 5 min. Clear supernatants were transferred into new tubes and stored at −80 °C. The concentrations of interleukin 6 (IL-6), interleukin 8 (CXCL8), monocyte chemoattractant protein-1 (MCP-1/CCL2), tumor necrosis factor-α (TNF-α), growth regulated oncogene-α (Gro-α/CXCL1), and growth regulated oncogene-β (Gro-β/CXCL2) were quantified by the Luminex-based multiplex technique according to the manufacturer’s instructions (Bio-Rad, Hercules, USA). Standard curves and concentrations were calculated using the Bio-Plex Manager 6.0 with protein detection sensitivity between 1 pg/mL and 40 µg/mL. All samples were measured as triplicates.

### 2.8. Statistical Analysis

The cell numbers were statistically analyzed using GraphPad Prism 8 (GraphPad Software, San Diego, CA, USA). Statistical significance was determined using the two-way ANOVA with Sidak’s multiple comparisons test. Data points are represented as Box and Whiskers with Tukey error bars.

The statistical analysis of cytokine measurements was performed by GraphPad Prism 7 (GraphPad Software, San Diego, CA, USA). The normal distribution was tested by applying the D’ Agostino–Pearson normality test. The statistical significance was determined using the Holm–Sidak method and data were presented as Box and Whiskers with Tukey error bars.

## 3. Results

### 3.1. Cell Morphology, Vitality, and Attachment of HGF were Rapidly Impaired After Challenge with the S. oralis Biofilm, whereas HGEp Remained Unaffected

**Figure 2:**
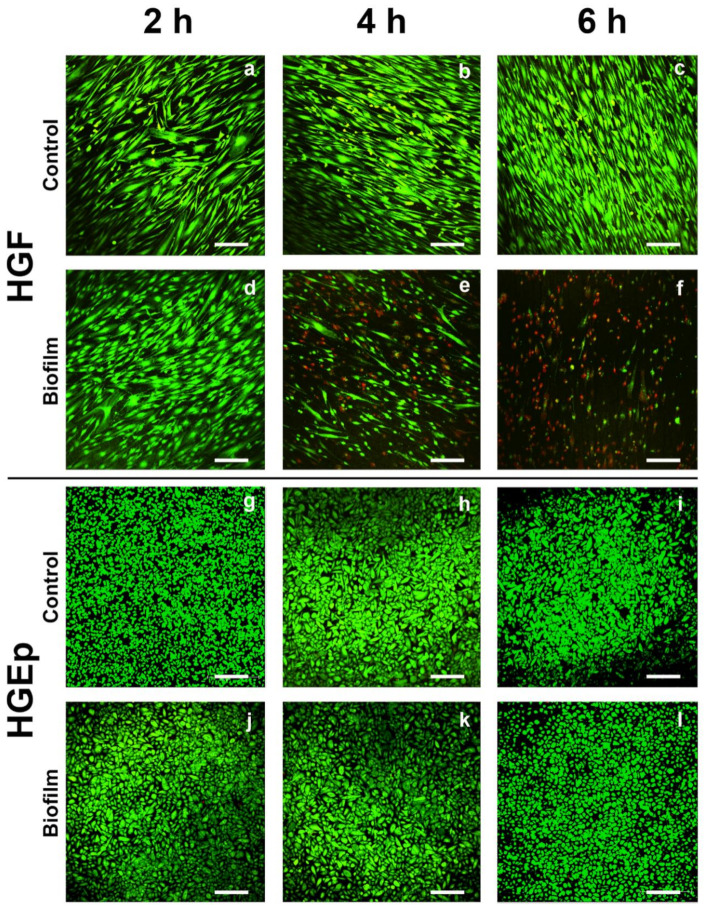
The vitality of gingival tissue cells after the *S. oralis* biofilm challenge. Tissue cells grown on titanium disks were co-cultured with the *S. oralis* biofilm or a control membrane for up to 6 h. Subsequently, the cells were stained for LIVE/DEAD. Green depicts live cells and red dead cells. The HGF remained vital in the control samples after 2, 4, or 6 h—(**a**), (**b**), and (**c**), respectively. After 2 h of co-culture with the *S. oralis* biofilm, the vitality of HGF was similar to that of the control (**d**). Many dead cells were detected after 4 h (**e**) and almost all were dead after 6 h (**f**). The HGEp remained vital in the control samples after 2, 4, or 6 h—(**g**), (**h**), and (**i**), respectively. The HGEps remained unaffected after co-culture with the *S. oralis* biofilm for 2, 4, or 6 h—(**j**), (**k**), and (**l**), respectively. Typical pictures from four to five independent experiments. Scale bars: 200 µm.

The effects of the *S. oralis* biofilm on the cell morphology and vitality of the tissue cells were evaluated by qualitative LIVE/DEAD staining and microscopic examination. The cell morphology of the HGF was similar to the control cells after 2 h biofilm challenge (Figure 2a,d). After 4 h, most cells lost their fibroblastic morphology, and they were rounded and dead according to the staining (Figure 2e). After 6 h, almost all cells were dead and many were detached from the titanium surface (Figure 2f) compared to the control (Figure 2c). The morphology and vitality of the biofilm-challenged HGEps were similar to those of the control cells at all time points (Figure 2g–l). The co-culture of HGF with the *S. oralis* biofilm, initiated cell death and a change in the cell morphology after only 4 h. In contrast, the vital status and morphology of HGEps remained unaffected during co-culture with the *S. oralis* biofilm.

**Figure 3:**
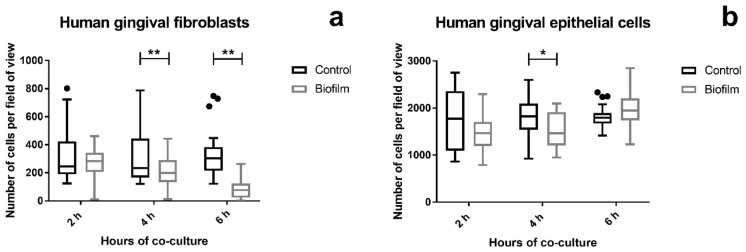
Cell attachment of gingival tissue cells after *S. oralis* biofilm challenge. Tissue cells grown on titanium disks were co-cultured with the *S. oralis* biofilm or a control membrane for up to 6 h. The cells were fixed after examination of the LIVE/DEAD staining and their nuclei were stained with 4′,6-Diamidino-2-phenylindole dihydrochloride (DAPI). Five random images were acquired from each sample and nuclei were automatically counted using ImageJ. Cell attachment of HGF decreased over time in response to the *S. oralis* biofilm (**a**). In HGEp, the cell attachment remained mainly unaffected, except for a slight decrease in cell numbers after 4 h of co-culture with the biofilm (**b**). Statistical significance was determined using the two-way ANOVA with Sidak’s multiple comparisons test. Data points are represented as Box and Whiskers with Tukey error bars. Asterisks indicate *p* < 0.05 and double asterisks *p* < 0.01. HGFs: data from two independent experiments (six control cultures and six co-cultures). HGEps: data from two independent experiments (five control cultures and five co-cultures). HGF 4 h: *p* = 0.0057; HGF 6 h: *p* < 0.0001; HGEp 4 h: *p* = 0.04442.

Changes in cell attachment on titanium disks were quantified by staining and counting the cell nuclei after microscopic examination of the LIVE/DEAD stained tissue cells. The cell attachment of HGF significantly decreased after 4 h of co-culture with the *S. oralis* biofilm compared to the control. This phenomenon was more prominent after 6 h (Figure 3a and Figure S1). In contrast, the cell attachment of HGEp was mostly the same in all groups. Only after 4 h challenge with the biofilm, slightly fewer HGEps were counted (Figure 3b and Figure S2), which could be due to fluctuations in confluency. The co-culture of the *S. oralis* biofilm induced cell attachment of the HGF over time, while HGEp remained unaffected.

It was indicated by the qualitative LIVE/DEAD staining that HGF detached from titanium disks in parallel to the raising cell death over time. This observation was then confirmed by quantitative cell counting.

### 3.2. Strong Transcriptional Response to Commensal S. oralis biofilm in HGF Compared to HGEp

**Figure 4:**
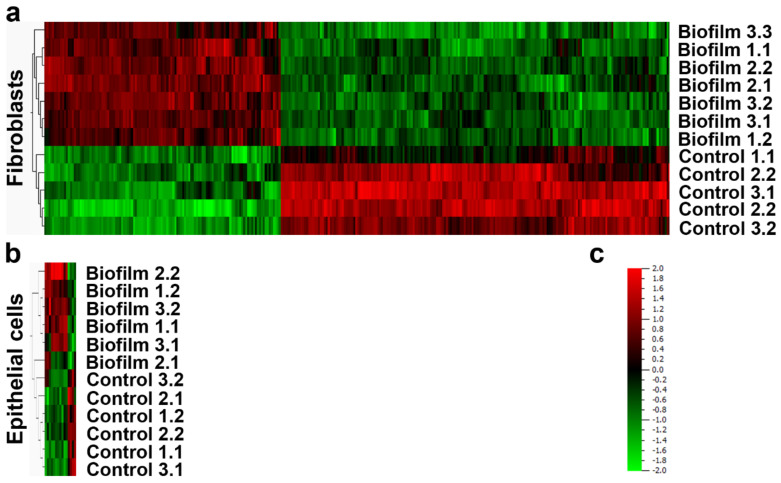
Transcriptional analysis of peri-implant tissue cells after 2 h challenge with *S. oralis* biofilm. The HGFs (**a**) showed a broader response than the HGEps (**b**). The heat-maps were generated using Qlucore Omics Explorer 3.2 after the hierarchical clustering of the experimental groups and the differentially regulated genes (data from three independent experiments). The intensity of red indicates the upregulation of genes after co-culture and intensity of green downregulation (**c**). Detailed gene lists of upregulated and downregulated genes are recorded in the supplementary material (Tables S3–S6). HGF: data from three independent experiments (five control cultures and seven co-cultures). HGEp: data from three independent experiments (six control cultures and six co-cultures).

The RNA of HGFs was already hydrolyzed to a large extent after 4 h of co-culture (Table S1). Therefore, the gene expression analysis through microarrays was performed after 2 h, to retain adequate RNA quality (Table S2). After the biofilm challenge, 302 genes were differentially regulated in HGFs (Figure 4a and Tables S3 and S4). Pathways related to inflammation, immune response, cell-cycle regulation, differentiation, apoptosis, development, and microbial detection were enriched after pathway analysis of the upregulated genes (Table 1). Despite the high number of downregulated genes in the biofilm group, no pathway enrichment was observed—indicating that those genes have extremely diverse functions. Transcriptional analysis of HGEps resulted in 15 regulated genes (Figure 4b and Tables S5 and S6). All upregulated genes in the biofilm group were related to inflammation or the immune response (Table 1). Pathway enrichment of the downregulated genes was not observed for HGEps either.

After comparing transcriptional analysis between the investigated cell types, we observed that the number of genes regulated in HGF was 20 times more than in HGEp (Figure 4). The common enriched pathways in HGF and HGEp were related to proinflammatory and the immune response, including genes of proinflammatory factors, *IL6*, *CCL2*, *CXCL8*, *CXCL1,* and *CXCL2* (Table 1). Also, the upregulated genes in HGF included genes encoding ligands, receptors, transcription factors, and inductive or inhibitory signal transducers of related pathways, whereas the activity of upregulated genes in HGEp is arranged only at the beginning and the end of each pathway (Table 1). Overall, the transcriptional response of HGEps to the *S. oralis* biofilm was narrow and focused on the inflammatory response without indication for regulation within the enriched pathways. In contrast, HGFs expressed many additional genes, which could participate in the downstream regulation of the enriched pathways. These genes might lead to diverse cell functions including cell-cycle regulation, apoptosis, differentiation, and development. The directionality of whole pathways was not indicated since this requires further investigation with proteomic data. Even so, pathway analysis allowed the conversion of gene expression data into biological meaningful data, which revealed regulated biological processes and molecular functions after *S. oralis* biofilm challenge.

### 3.3. Attenuation of Proinflammatory Cytokines in HGF and HGEp after Their Co-Culture with S. oralis Biofilm

**Figure 5:**
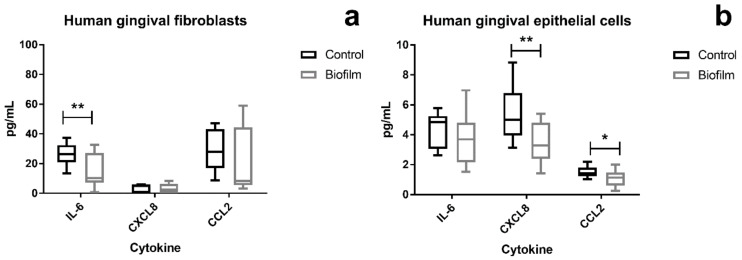
Levels of secreted proinflammatory cytokines from peri-implant tissue cells after 2 h co-culture with the *S. oralis* biofilm. Cytokines were measured using a Bio-Plex Kit for multiplex analysis. In HGFs, interleukin 6 (IL-6) levels were significantly reduced after co-culture (**a**). The interleukin 8 (CXCL8) and CCL2 levels were significantly reduced in HGEps after biofilm challenge (**b**). Data were tested for normal distribution (D’Agostino–Pearson normality test) and statistical significance was determined using the multiple T-test with corrections for multiple comparisons using the Holm–Sidak method, when *p* < 0.05. Data points are represented as Box and Whiskers with Tukey error bars. Asterisks indicate *p* < 0.05 and double asterisks *p* < 0.01. HGFs: data from three independent experiments (five control cultures and seven co-cultures). HGEps: data from three independent experiments (six control cultures and six co-cultures). HGF IL-6: *p* = 0.02684; HGEp CXCL8: *p* = 0.001608; HGEp CCL2: *p* = 0.01979.

Genes of proinflammatory factors were upregulated in both cell types after biofilm challenge, and the protein levels, which were secreted into the co-culture supernatants, were measured. The levels of IL-6, CXCL8, MCP-1/CCL2, TNF-α, Gro-α/CXCL1, and Gro-β/CXCL2 that can be secreted by the tissue cells were measured using the Luminex technology. The levels of IL-6, CXCL8, and CCL2 were in the detectable range of the cytokine assay (Bio-Plex Pro Human Chemokine 6plx EXP, Bio-Rad Laboratories, Inc., US) (Figure 5). In HGFs, the IL-6 levels were significantly lower after 2 h *S. oralis* biofilm challenge (Figure 5a), whereas the levels of CXCL8 and CCL2 were significantly lower in HGEps than in the control group (Figure 5b).

## 4. Discussion

Dental implants are surrounded by mucosal soft tissue [6,55]. The major cell types in soft tissue are HGEps and HGFs. The HGEps face the oral cavity, whereas HGFs are located within the connective tissue. HGEps are subsequently in constant contact with oral biofilms, whereas HGFs are protected by the epithelial layer until the tissue of the epithelium is injured [7,9,10,56]. Previous studies indicated that oral commensal streptococci protect tissue [16,41,42,43]. We compared the response of HGEps and HGFs in a peri-implant microenvironment to commensal *S. oralis*, which can be present in high numbers at implant sites in oral health [21,57]. HGFs and HGEps showed distinct responses to the *S. oralis* biofilm and we concluded that *S. oralis* can only protect tissue when the peri-implant mucosa is intact.

The cell morphology and vitality of HGFs were rapidly impaired after challenge with an *S. oralis* biofilm (Figure 2d–f). In contrast, the morphology and vitality of HGEps remained unaffected (Figure 2j–l). Despite this difference, both cell types probably sensed the presence of *S. oralis*, as indicated by the transcriptional analysis, where pathways involved in bacteria recognition—such as toll-like receptor (TLR) and nucleotide-binding oligomerization domain (NOD)-like receptor signaling—were enriched (Table 1). These pathways are involved in bacteria recognition, mainly through the pathogen recognition receptors (PRRs) [58,59], which may sense the peptidoglycans in the cell wall of *S. oralis* [58]. It was previously shown that HGEps and HGFs constitutively express PRRs (i.e., TLR1 to 9 as well as NOD1 and NOD2) [60,61]. Any differential expression of PRRs was not observed after the analysis of gene expression (Tables S3–S6). Pathways related to the inflammatory cell response were enriched in both cell types. In HGFs, additional genes and pathways were upregulated, and these were related to cell-cycle regulation, apoptosis, development, and differentiation. Moreover, more genes were differentially regulated than in HGEps (Figure 4). More genes were regulated within enriched pathways in HGFs (Table 1) indicating a more intense response to the *S. oralis* biofilm. Besides, regulated genes in HGF are distributed within the pathways, whereas regulated genes in HGEps are almost only at the beginning and end of each pathway (Table 1 and Figures S4–S19). Thus, we assume that pathway regulations are more probable in HGF. Our results highlight the susceptibility of HGFs, which exhibited morphological and transcriptional stress responses. The HGEps exhibited an inflammatory response to the *S. oralis* biofilm, but their cell morphology and vitality were not affected. This difference in response between HGFs and HGEps might emerge from their distinct tissue topography. HGEp topography at tissue borders allowed host adaptation since the cells were in constant contact with the highly abundant commensal bacteria [62]. Belibasakis et al. showed that the transcriptional response of HGF is not specifically dependent on the composition of subgingival biofilms. The HGF response was similar to biofilms, unrelated to whether they included the three ‘red complex’ pathogens (i.e., *Porphyromonas gingivalis, Treponema denticola,* and *Tannerella forsythia*) or not [5,10]. This observation also underlines the lack in HGFs of adaptive coevolution with oral bacteria, which are protected within the connective tissue [7]. Another group showed that the transcriptional response of HGEps to the commensals *Streptococcus gordonii* and *Fusobacterium nucleatum* is similar; however, it differs from the response to the pathogens *Aggregatibacter actinomycetemcomitans* and *P. gingivalis [16,63]*. This indicates that HGEps are probably able to distinguish between bacterial species and highlights their coevolution with bacteria. Our study presents a direct comparison between HGFs and HGEps. The results of this comparison revealed that the HGFs were more susceptible to challenge with the *S. oralis* biofilm than were HGEps. This may be linked to their different topographies relative to soft tissue—with HGEp being located at the epithelial layer facing the oral cavity and HGFs underneath and within the connective tissue.

As previously discussed, genes of proinflammatory factors were upregulated in both cell types after biofilm challenge. The corresponding protein levels in the co-culture supernatants were not in line with the upregulated gene expression. Instead, IL-6 was lower after challenge with the *S. oralis* in HGFs and CXCL8 was lower in HGEps (Figure 5). In addition, CCL2, which was not regulated transcriptionally, was lower in HGEps. During the steady-state of a cell, the protein levels normally have an ideal correlation to the mRNA levels, however, during differentiation or stress response this correlation might be disrupted [64]. Cytokine production is not only regulated during transcription but also post-transcriptionally through mRNA stabilization or decay [65]. Another checkpoint for cytokines is the modulation of storage and secretion [66]. So, it is not surprising that the elevated mRNA levels of CXCL8, IL-6, and CCL2 after biofilm challenge are not necessarily associated with increased protein levels.

Attenuation of proinflammatory cytokines by *S. oralis* might support tissue protection. Basso et al. demonstrated that high levels of IL-6 and CXCL8 affect directly HGFs and HGEps. These factors induce apoptosis and decrease cell migration in both gingival cell types, this negative effect on their mucosal healing properties was more evident in HGFs [67], although they express low levels of IL-6 receptor [68]. Besides that direct effect, high levels of proinflammatory factors also activate and recruit immune cells, which can, in turn, promote tissue destruction [56,69,70]. Decreased IL-6 and CXCL8 levels were also detected in HGEps after co-culture with *S. gordonii*, whereas pathways leading to secretion of proinflammatory factors were enriched (i.e., mitogen-activated protein kinase (MAPK) signaling pathway and TLR pathway) [16]. This observation from Hasegawa et al. was in line with the transcriptional analysis (Table 1) and secreted cytokine levels in response to *S. oralis* (Figure 5). It has been shown that other streptococci (i.e., *Streptococcus salivarius* K12 and *Streptococcus cristatus*) attenuate CXCL8 levels too, even after stimulation with *F. nucleatum*, which induces CXCL8 [41,42,43]. It is also noteworthy that the mRNA of CXCL8 was not regulated after bacterial challenge in these studies [41,42,43]. It has been proposed that commensal species can modulate the host response to become hyporesponsive; thereby, they could limit tissue destruction induced by pathogenic bacteria [16,54]. A clinical study reported reduced CXCL8 levels and improved clinical appearance in patients with mild oral inflammation after the use of chewing gums containing *Lactobacillus reuteri* [71]. Furthermore, the clinical application of *S. salivarius* 24SMB and *S. oralis* 89a was effective against recurrent acute otitis media and recurrent acute pharyngotonsillitis [72,73]. These strains can inhibit biofilm formation by upper respiratory tract pathogens [40]. Similar biofilm inhibition against periodontopathogens has also been shown for other species [74]. Commensal streptococci seem to exert tissue-protective properties, although different commensal species may induce distinct immune responses in host cells—which may explain differences between species in cell response [41].

The values for cytokine secretion suggest that *S. oralis* biofilm might protect tissue; however, in HGFs transcriptional inflammatory and stress responses were induced as well as a loss of cell vitality and attachment. Biofilm challenge also induced a transcriptional inflammatory response in HGEps, but the overall condition and attachment of these cells remained unaffected and there was no additional stress response linked to apoptosis. The *S. oralis* biofilm induced a transcriptional inflammatory response in both cell types, in contrast to the changes in secreted proinflammatory factors, which were in the opposite direction. Since HGFs were rapidly damaged—as also reflected in the RNA quality (Table S1)—a direct comparison to HGEp was limited to the 2 h response. Cell vitality and attachment of HGFs were dramatically impaired after 6 h of co-culture (Figure 2). Considering this limitation for HGFs, the cell condition of HGEps was also investigated up to 6 h for a direct comparison. The HGEps may exhibit a comparable response to HGF at time points later than 6 h, if we bear in mind that the exposure of monolayers to biofilms differs from that of epithelial tissue. In another study, where HGFs and HGEps were stimulated with LPS (lipopolysaccharide), which allows longer periods, the viable HGFs decreased already after 24 h, whereas the HGEps after 48 h indicating a delayed response [75]. Nevertheless, even delayed response of HGEp supports the conclusion that HGFs are more susceptible than HGEps and their initial responses to the *S. oralis* biofilm differ. Stimulation of HGEps with synthetic TLR (toll-like receptor) and NOD agonists did not lead to increased secretion of CXCL8, IL-6, and CCL2. Instead, activation of TLRs and NODs induced the expression of antibacterial PGRPs (peptidoglycan recognition proteins) [59,76]. By that mechanism, HGEps probably prevent bacterial invasion without intense inflammatory response, which might cause tissue destruction [59,76]. In contrast, stimulation of HGFs resulted in the augmentation of CXCL8, IL-6, and CCL2 [60]. Since HGEps are in constant contact with the normal oral flora, it is justified that they do not secrete proinflammatory factors with potential tissue destructive action. HGFs secreted proinflammatory factors upon stimulation with microbial components. Normally, HGFs are isolated from normal flora; however, in the case of tissue injury, they will interact with oral bacteria [60]. We did not detect such a discrepancy in our study. This could be attributed to the use of the *S. oralis* biofilm with potential proteolytic activity [77,78,79], instead of synthetic microbial components or it could be that the surface of the titanium disk also affects the cytokine secretion. It has been shown that cytokine secretion might change in response to different implant surfaces [80]. Nonetheless, the general observation that HGFs react more intense than HGEps to the biofilm challenge is following previous comparisons between these cell types. Even though IL-6 is attenuated, the impact of *S. oralis* is prominent on HGFs especially at later time points, where cells die and detach from the titanium disk. On the one hand, the lower IL-6 levels most probably would affect the surrounding tissue avoiding further tissue destruction by other host cells. On the other hand, the cell detachment and death of HGFs would destroy the connective tissue in response to *S. oralis*. Our results indicate the tissue-protective properties of *S. oralis*, which are reflected in its full extent in HGEps, which were not damaged by the *S. oralis* biofilm during the observed timeframe.

To our knowledge, this is the first study showing the direct comparison of HGFs and HGEps in response to a biofilm. The results indicate that the *S. oralis* biofilm has a protective role on the peri-implant tissue borders and highlights the adaptive co-evolution of epithelial cells with oral commensal bacteria; however, if the underlying connective tissue is exposed, *S. oralis* can affect the HGFs, which are susceptible. This could induce further tissue destruction. Overall, the results underline the importance of an intact implant–epithelium interface and indicate that any lesions may lead to the initial response of tissue destruction by HGFs in connective tissue, even if pathogens are absent. These cell-specific responses should be considered during the in vitro investigation of host–pathogen interactions. Future studies using in vitro systems including both cell types could provide further details.

## 5. Conclusions

We could gain new insights into the response of the two main cell types of the peri-implant mucosa to the commensal *S. oralis,* a highly abundant species on healthy dental implants [3,4,5,6,20,21,22,23]. HGEps—which are in constant contact with the *S. oralis*—exhibited a subtle reaction without impact on their cell condition and attachment. In contrast, HGF, which is protected by the epithelial layer under physiological conditions, was highly susceptible. Our results provide evidence that an intact soft-tissue seal at the implant is required to maintain health and is a prerequisite for a protective effect of commensal biofilms, which can promote homeostasis. Our findings might support future guidelines for the prevention and treatment of peri-implant disease.

## Figures and Tables

**Table 1:** Enriched pathways of upregulated genes from HGFs and HGEps after challenge with the *S. oralis* biofilm for 2 h. The potential role of each gene in the signal transduction of respective pathways is indicated.

	Human Gingival Fibroblasts			Human Gingival Epithelial Cells		
**Pathway**	**%**	***p***	**Genes**	%	*p*	Genes
Chemokine signaling pathway	5.32	0.0421	CXCL1^L^, CCL2^L^, NCF1^RS^, CXCL3^L^, CXCL2^L^	0.25	0.0068	CXCL1^L^, CXCL2^L^, CXCL8^L^
Cytokine-cytokine receptor interaction	5.32	0.0791	CSF2^L^, IL6^L^, CCL2^L^, IL12A^L^, TNFSF9^L^	0.25	0.0103	IL6^L^, TNF^L^, CXCL8^L^
FoxO signaling pathway	7.45	0.0003	SGK1^TI^, IL6^L^, PLK3^RS^, PLK2^RS^, GADD45G^RS^, GADD45B^RS^, KLF2^RS^			
Jak-STAT signaling pathway	5.32	0.0190	CSF2^L^, IL6^L^, SOCS3^TI^, IL12A^L^, MYC^TF^			
MAPK signaling pathway	10.64	0.0001	DUSP5^TI^, FGF5^L^, FOS^TF^, DUSP2^TI^, DUSP1^TI^, JUN^TF^, GADD45G^TA^, GADD45B^TA^, MYC^TF^, NGF^L^			
NF-kappa B signaling pathway				0.17	0.0614	TNF^L^, CXCL8^RS^
NOD-like receptor signaling pathway				0.25	0.0006	IL6^RS^, TNF^RS^, CXCL8^RS^
p53 signaling pathway	3.19	0.0814	GADD45G^TI, RS^, PMAIP1^TA^, GADD45B^TI, RS^			
PI3K-Akt signaling pathway	6.38	0.0959	FGF5^L^, SGK1^TA^, IL6^L^, MYC^TF^, EPHA2^RC^, NGF^L^			
TNF signaling pathway	11.70	0.0000	CXCL1^RS^, CSF2^RS^, FOS^TF, RS^, IL6^RS^, CCL2^RS^, SOCS3^RS^, CXCL3^RS^, JUN^TF, RS^, EDN1^RS^, CXCL2^RS^, JUNB^TF,RS^	0.34	0.0000	CXCL1^RS^, IL6^L^, TNF^L^, CXCL2^RS^
Toll-like receptor signaling pathway	4.26	0.0389	FOS^TF^, IL6^RS^, JUN^TF^, IL12A^RS^	0.25	0.0023	IL6^RS^, TNF^RS^, CXCL8^RS^
Wnt signaling pathway	4.26	0.0739	DKK1^TI^, JUN^TF^, BAMBI^TI^, MYC^TF^			

Empty cells indicate no pathway enrichment for the cell type. Only relevant pathways for *S. oralis* cell response were included in the table (see Table S7). Role of genes in respective pathway is depicted by: L = ligand; RS = response to pathway activation; TI = transducer for inhibition; TA = transducer for activation; TF = transcription factor; RC = receptor. % = is calculated by the number of involved genes from the total number of human genes. p = is calculated from the number of genes present in each pathway in relation to the number of all regulated genes, which were submitted to DAVID.

## Supplementary Materials

The following are available online at https://www.mdpi.com/2073-4409/9/5/1226/s1, Table S1: Quality of RNA isolated from human gingival fibroblasts after 2, 4, or 6 h of co-culture with the *S. oralis* biofilm, Table S2: Quality of RNA isolated from human gingival fibroblasts and epithelial cells after 2 h of co-culture with the *S. oralis* biofilm, Table S3: List of upregulated genes in human gingival fibroblasts after 2 h *S oralis* biofilm challenge, Table S4: List of downregulated genes in human gingival fibroblasts after 2 h *S. oralis* biofilm challenge, Table S5: List of upregulated genes in human gingival epithelial cells after 2 h *S. oralis* biofilm challenge, Table S6: List of downregulated genes in human gingival epithelial cells after 2 h *S. oralis* biofilm challenge, Table S7: List of all enriched pathways in fibroblasts and epithelial cells after 2 h *S. oralis* biofilm challenge, Figure S1: Cell nuclei of HGFs on titanium disks after *S. oralis* biofilm challenge, Figure S2: Cell nuclei of HGEps on titanium disks after *S. oralis* biofilm challenge, Figure S3: Effect of cell culture media on *S. oralis* biofilm volume and vitality, Figures S4-S11: Role of differentially regulated genes in HGF and HGEp within common enriched pathways, Figures S12-S17: Role of differentially regulated genes in HGF within the enriched pathways, Figures S18-S19: Role of differentially regulated genes in HGEp within the enriched pathways.
