# Supplementary material for: In Vitro Effects of Streptococcus oralis Biofilm on Peri-Implant Soft Tissue Cells
Source: Cells. 2020 May 15;9(5):1226. doi: 10.3390/cells9051226 (PMC7290395; doi:10.3390/cells9051226)
Supplement: Supplementary file 1 [file cells-09-01226-s001.pdf]

SUPPLEMENTARY MATERIAL

In vitro effects of *Streptococcus oralis* biofilm on peri-implant soft-tissue cells

Alexandra Ingendoh-Tsakmakidis [1,*], Jörg Eberhard [1,3], Christine S. Falk [2], Meike Stiesch [1] and Andreas Winkel [1]

[1] Department of Prosthetic Dentistry and Biomedical Materials Science; Hannover Medical School, Carl-Neuberg-Str. 1, 30625 Hannover, Germany
[2] Institute of Transplant Immunology; Hannover Medical School, Carl-Neuberg-Str. 1, 30625 Hannover, Germany
[3] Current Address: University of Sydney, Sydney Dental School, 2 Chalmers St, Westmead NSW 2010, Australia
* Correspondence: ingendoh-tsakmakidis.alexandra@mh-hannover.de

Supplementary Materials and Methods

The quality and quantity of isolated total RNA was determined using the Agilent 2100 Bioanalyzer according to the manufacturer's protocol

Gene lists generated after analysis with the Qlucore Omics Explorer, were submitted to Pathview (pathway based data integration and visualization) (Luo W, Pant G, Bhavnasi YK, Blanchard SG, Brouwer C. Pathview Web: user friendly pathway visualization and data integration. Nucleic Acids Res, 2017, Web Server issue, doi: 10.1093/ nar/gkx372; Luo W, Brouwer C. Pathview: an R/Biocondutor package for pathway-based data integration and visualization. Bioinformatics, 2013, 29(14):1830-1831, doi: 10.1093/bioinformatics/btt285) in order to present gene expression data on graphs of the enriched pathways. The gene lists together with the values of difference in expression from HGF and HGEp were submitted separately. In addition, the lists of enriched pathways, which resulted from analysis using DAVID (ref), were also submitted to Pathview for each cell type.

Supplementary Results

Quality of isolated RNA from tissue cells in control and biofilm groups

Table S1. Quality of RNA isolated from human gingival fibroblasts after 2, 4, or 6 hours of co-culture with the *S. oralis* biofilm.

	**RNA integrity number (RIN)**	
**Co-culture time**	Control	Biofilm
**2 hours**	9	8.5
**4 hours**	9.9	6.1
**6 hours**	8.4	5.8

Table S2. Quality of RNA isolated from human gingival fibroblasts and epithelial cells after 2 hours of co-culture with the *S. oralis* biofilm. Samples used for gene expression analysis.

**Human gingival fibroblasts**		**Human gingival epithelial cells**	
**Sample**	RIN	Sample	RIN
**Control 1.1**	8.6	Control 1.1	10
**Biofilm 1.1**	8.2	Control 1.2	9
**Biofilm 1.2**	7.5	Biofilm 1.1	9.4
**Control 2.1**	9.6	Biofilm 1.2	9.5
**Control 2.2**	7.8	Control 2.1	9.8
**Biofilm 2.1**	8.1	Control 2.2	10
**Biofilm 2.2**	8.8	Biofilm 2.1	9.
**Control 3.1**	9.7	Biofilm 2.2	8.7
**Control 3.2**	9.7	Control 3.1	9.9
**Biofilm 3.1**	7.7	Control 3.2	9.7
**Biofilm 3.2**	7.8	Biofilm 3.1	10
**Biofilm 3.3**	7.7	Biofilm 3.2	10

Lists of differentially regulated genes in HGFs after S. oralis biofilm challenge

Table S3. List of upregulated genes in HGFs after 2 hours *S oralis* biofilm challenge.

Gene name	Systematic name	p	Fold change	log2 difference
**HAS2**	NM_005328	9.88E+11	0.382783	1.385401913
**FOS**	NM_005252	5.41E+09	0.021441	5.543471366
**DUSP1**	NM_004417	2.98E+09	0.204405	2.290499958
**RGS2**	NM_002923	1.95E+11	0.441866	1.178319526
**NGF**	NM_002506	0.002184	0.403271	1.310178257
**KLF2**	NM_016270	2.09E+09	0.275347	1.860675518
**PTX3**	NM_002852	2.76E+11	0.490559	1.027501925
**C5orf30**	NM_033211	4.74E+10	0.454952	1.13621441
**S100A14**	NM_020672	2.61E+11	0.359811	1.474688529
**AQP10**	NM_080429	0.000124	0.337651	1.566396987
**ADM**	NM_001124	5.59E+10	0.294003	1.766096438
**SNAI1**	NM_005985	0.000259	0.45946	1.12198867
**SIK1**	NM_173354	0.007908	0.251556	1.991050834
**CSF2**	NM_000758	4.93E+10	0.151731	2.720409818
**ID3**	NM_002167	1.25E+11	0.354185	1.497425404
**KRTAP1-1**	NM_030967	0.000236	0.423329	1.240147974
**EPHA2**	NM_004431	3.03E+11	0.425327	1.233354735
**DUSP5**	NM_004419	0.001637	0.380686	1.393325057
**AMOTL2**	NM_016201	1.26E+09	0.314793	1.667525496

**RBM24**	NM_153020	0.000114	0.415854	1.265851891
**MAFB**	NM_005461	0.001049	0.475464	1.072591301
**CTGF**	NM_001901	0.000454	0.273796	1.868825508
**SGK1**	NM_005627	0.000173	0.38038	1.394487813
**SOCS3**	NM_003955	3.58E+09	0.320188	1.643007711
**FGF5**	NM_004464	1.77E+11	0.476314	1.070015568
**EGR1**	NM_001964	0.004469	0.213632	2.226797291
**EDN1**	NM_001955	0.000304	0.207265	2.270450306
**CITED2**	NM_006079	9.60E+10	0.397261	1.33184084
**MYC**	NM_002467	6.02E+09	0.163676	2.611084438
**RGS16**	NM_002928	1.65E+11	0.090682	3.463041047
**DKK1**	NM_012242	7.85E+08	0.212383	2.235259083
**PLK2**	NM_006622	8.02E+09	0.277368	1.850127626
**CXCL2**	NM_002089	0.002368	0.18088	2.46689369
**RGS16**	NM_002928	5.93E+10	0.088819	3.492988393
**ARC**	NM_015193	0.000259	0.328544	1.605841407
**HIST4H4**	NM_175054	5.79E+11	0.384887	1.377493898
**ZFP36**	NM_003407	2.50E+11	0.432125	1.210480084
**HIST1H4E**	NM_003545	0.000217	0.194982	2.358585551
**MT1E**	XM_005255956	2.46E+10	0.246194	2.022133858
**HIST1H4A**	NM_003538	0.001586	0.413369	1.27449883
**CYR61**	NM_001554	2.46E+11	0.073352	3.769029088
**EGR2**	NM_000399	0.005119	0.320845	1.640050488
**HHEX**	NM_002729	0.00091	0.393783	1.344526928
**KCTD4**	NM_198404	1.68E+11	0.269334	1.892529718
**PLK3**	NM_004073	7.96E+10	0.443934	1.171584169
**BAMBI**	NM_012342	0.000654	0.473096	1.079795455
**YRDC**	NM_024640	1.35E+10	0.497683	1.00669981
**IL6**	NM_000600	5.55E+10	0.06041	4.049065147
**CXCL1**	NM_001511	2.82E+11	0.24464	2.031265011
**CH25H**	NM_003956	0.005659	0.47507	1.073789259
**CCL2**	NM_002982	9.02E+11	0.302539	1.724804732
**IL12A**	NM_000882	1.67E+10	0.340828	1.552882459
**CAMK1G**	NM_020439	0.004734	0.41882	1.255599012
**HBEGF**	NM_001945	7.75E+09	0.137862	2.858702659
**BU963192**	BU963192	0.000195	0.451813	1.146202843
**CXCL3**	NM_002090	4.83E+11	0.182483	2.454167179
**RNU12**	NR_029422	1.55E+11	0.105308	3.247310659
**ANKRD37**	NM_181726	0.000559	0.437629	1.1922199
**GADD45B**	NM_015675	1.07E+10	0.193766	2.367610894
**JUNB**	NM_002229	0.002233	0.305398	1.71123543

**TRIB1**	NM_025195	0.004115	0.499263	1.002127015
**CXCL2**	NM_002089	7.55E+11	0.233587	2.09796693
**SNHG7**	NR_003672	9.36E+10	0.409629	1.287609235
**DUSP2**	NM_004418	0.000216	0.170917	2.548635935
**lnc-EIF2D-1**	lnc-EIF2D-1:1	1.37E+09	0.24567	2.025205843
**IER2**	NM_004907	4.43E+11	0.400609	1.319735027
**SCARNA17**	NR_003003	3.28E+10	0.183274	2.44792748
**CITED2**	NM_006079	0.001596	0.420152	1.251015998
**A_33_P3216635**	N/A	0.001576	0.434635	1.202124387
**MAFB**	NM_005461	0.003991	0.461305	1.116207469
**A_33_P3223860**	N/A	0.000422	0.481083	1.055641186
**SNORD3B-1**	NR_003271	7.77E+08	0.052537	4.250529791
**F3**	NM_001993	9.95E+11	0.337466	1.567185169
**A_33_P3227225**	N/A	4.70E+11	0.365531	1.451932693
**BF515046**	BF515046	3.89E+10	0.318425	1.650972597
**LOC100131831**	AK129685	7.25E+11	0.445451	1.166661864
**SNORD3B-1**	NR_003271	2.85E+09	0.082341	3.602252784
**GADD45G**	NM_006705	0.005489	0.496825	1.009191247
**AK127417**	AK127417	6.24E+10	0.374447	1.417168101
**HIST2H3A**	NM_001005464	0.000342	0.3083	1.697593959
**EDN1**	NM_001955	0.000558	0.248585	2.00818798
**PTGES2-AS1**	NR_024425	4.44E+10	0.395364	1.338746196
**WDR74**	ENST00000538098	9.63E+11	0.27061	1.885710372
**RNVU1-18**	NR_004400	1.23E+10	0.133612	2.903883908
**A_33_P3269528**	N/A	0.002235	0.466229	1.100889836
**SCARNA2**	NR_003023	6.96E+08	0.22407	2.157976402
**LINC01347**	NR_029401	0.000492	0.478987	1.061942106
**RNU2-1**	NR_002716	7.02E+09	0.161047	2.634449677
**SNORD116-19**	NR_001290	1.44E+11	0.341702	1.549191231
**lnc-ATG2B-2**	lnc-ATG2B-2:1	2.40E+09	0.273891	1.868327478
**HOXA10**	NM_018951	0.000302	0.462853	1.111373514
**RHOB**	ENST00000272233	0.000105	0.492857	1.020758776
**KPRP**	NM_001025231	0.001408	0.335608	1.575152119
**A_33_P3317460**	N/A	7.01E+10	0.299464	1.739547153
**JUN**	NM_002228	0.000272	0.348336	1.521447851
**THC2492293**	THC2492293	8.65E+11	0.406263	1.29951287
**lnc-ZNF33A-2**	lnc-ZNF33A-2:1	5.48E+10	0.266121	1.909844445
**CXCL1**	NM_001511	4.54E+10	0.300343	1.735316877
**LINC00936**	NR_028138	0.001191	0.496884	1.009018869
**RNA28S5**	NR_003287	1.62E+11	0.466794	1.099141942
**NUAK2**	NM_030952	1.78E+11	0.316795	1.658379112

**THC2570839**	THC2570839	0.0017	0.479285	1.061044209
**A_33_P3351276**	N/A	3.01E+11	0.448869	1.155634621
**THC2605028**	THC2605028	0.004947	0.496012	1.011553411
**NCF1**	NM_000265	9.78E+10	0.458247	1.125803389
**RPPH1**	NR_002312	5.62E+09	0.1254	2.995393588
**RNA18S5**	NR_003286	0.002138	0.477303	1.067022697
**TNFSF9**	NM_003811	0.004402	0.446402	1.163584271
**RHOB**	ENST00000272233	0.003034	0.495559	1.012870897
**RNA5-8S5**	NR_003285	2.08E+09	0.154435	2.694928119
**RMRP**	NR_003051	0.000522	0.445248	1.167317781
**THC2610631**	THC2610631	0.000777	0.46705	1.098352328
**LOC51145**	XR_108997	2.73E+11	0.328416	1.60640451
**AK075182**	AK075182	0.008814	0.473871	1.077433834
**LOC729040**	ENST00000440769	1.32E+11	0.150754	2.729733191
**RNA28S5**	NR_003287	6.08E+09	0.235089	2.088721489
**PMAIP1**	NM_021127	0.001366	0.452891	1.142764913
**TTC34**	ENST00000579787	0.000274	0.374122	1.418419429
**LOC644656**	NR_036539	1.11E+11	0.323966	1.626086895
**RNU4ATAC**	DW419002	2.13E+07	0.012498	6.322121567

Table S4. List of downregulated genes in human gingival fibroblasts after 2 hours *S. oralis* biofilm challenge.

**Gene name**	**Systematic name**	**p**	**Fold change**	**log2 difference**
**RPL23AP32**	NR_002229	0.000954	2.15889525	-1.110293245
**SUN1**	ENST00000340926	0.002126	5.05566359	-2.337900468
**CYP1A1**	NM_000499	3.81E+11	3.47593546	-1.797401295
**TP53INP1**	NM_033285	0.001116	2.26221585	-1.177736591
**ZNF608**	NM_020747	0.001641	2.07679629	-1.054359711
**ACRC**	NM_052957	1.47E+11	2.28180909	-1.190178092
**NANOG**	NM_024865	1.70E+11	2.26603317	-1.180168979
**CYP1B1**	NM_000104	0.001446	2.44686651	-1.290935397
**AKAP12**	NM_144497	0.000732	2.32048917	-1.214428964
**DDX17**	NM_006386	0.000825	2.1100769	-1.077295578
**SMG1**	NM_015092	0.001303	2.19501948	-1.134233743
**ND6**	ENST00000361681	0.006892	2.1047225	-1.073630032
**MNT**	NM_020310	0.003126	2.12345171	-1.0864113
**IRF9**	ENST00000558468	6.51E+11	2.05145907	-1.03665037
**ZNF518A**	NM_014803	0.000534	2.13881731	-1.096813258
**UBXN7**	NM_015562	0.0003	2.05791044	-1.041180198
**ZMYND8**	ENST00000468376	0.002043	2.48970103	-1.31597251
**CIRBP**	ENST00000621399	0.000579	2.052737	-1.037548799
**BMF**	NM_001003940	0.000581	4.60692549	-2.203804265

**BCOR**	ENST00000615339	0.007912	2.9262135	-1.549035034
**PER2**	NM_022817	0.000643	2.18363047	-1.126728733
**ARSG**	ENST00000448504	0.000929	2.00072145	-1.000520322
**NKD2**	NM_033120	0.001831	2.03036332	-1.021737911
**CCDC149**	ENST00000324309	0.000623	2.51190472	-1.328781742
**LOC646652**	XR_427693	0.005487	2.29977345	-1.201491749
**KRTAP4-2**	NM_033062	0.004488	2.19721675	-1.135677195
**ZNF652**	NM_014897	0.00079	2.54058075	-1.34515832
**AGPAT9**	NM_032717	0.000264	2.34585047	-1.230111056
**AGPAT4-IT1**	NR_024277	0.000167	2.26200008	-1.17759898
**HIST1H1A**	NM_005325	0.001465	2.1732018	-1.119822147
**DPM3**	NM_018973	0.000159	2.04108787	-1.029338293
**PLEKHF1**	NM_024310	0.006258	2.01473904	-1.010592985
**RIPK4**	NM_020639	0.003928	3.08336568	-1.624506
**NPLOC4**	ENST00000374747	5.82E+11	3.15629387	-1.658231535
**SCAPER**	ENST00000303521	0.002297	2.25812101	-1.1751228
**KIAA1731NL**	NM_001243541	0.002869	2.56277609	-1.357707436
**ANKRD12**	NM_015208	0.000135	2.10337424	-1.072705562
**GHRLOS**	NR_073566	0.000938	2.31651187	-1.211954075
**ZMYM2**	ENST00000382881	0.005484	2.0298245	-1.021354996
**LRRC37A2**	NM_001006607	0.000378	2.0118742	-1.008540098
**WNT11**	NM_004626	0.001102	2.22566485	-1.154236362
**ETS2**	NM_005239	0.000208	2.13565636	-1.094679527
**KIAA0319L**	ENST00000485551	0.007486	2.75236797	-1.46067336
**SLC7A5**	NM_003486	0.003406	2.0060339	-1.004345986
**SLC1A2**	NM_004171	0.000428	2.04170227	-1.029772501
**ENST00000579793**	ENST00000579793	8.79E+10	2.04316831	-1.030808054
**DUSP5P1**	NR_002834	0.000789	4.41938829	-2.143846693
**PROSER2**	NM_153256	0.001035	2.16247177	-1.112681299
**AVIL**	NM_006576	4.60E+11	2.43493295	-1.283882046
**PROSER3**	NM_001039887	0.001828	2.17051482	-1.118037273
**RHOU**	NM_021205	0.000603	2.01941586	-1.013938037
**EPM2AIP1**	NM_014805	1.03E+11	2.20756054	-1.142453003
**FAM217B**	NM_022106	0.002532	2.10387897	-1.073051713
**RUNX1-IT1**	NR_026812	0.002015	2.67922306	-1.421814698
**MGEA5**	AF307332	7.44E+11	3.89478946	-1.961545343
**LOC344887**	NR_033752	0.008974	2.08120322	-1.057417845
**LOC100190986**	NR_024456	0.000848	4.2426796	-2.084975733
**SMA4**	NR_024054	0.000705	2.0562644	-1.040025782
**KIDINS220**	NM_020738	7.47E+11	2.2173295	-1.148823174
**AGAP9**	NM_001190810	0.000878	2.43373466	-1.283171886

**BC104430**	BC104430	9.31E+11	2.87922478	-1.525680424
**WDFY3**	ENST00000426414	0.001613	2.0461092	-1.032883143
**MALAT1**	NR_002819	0.00306	2.00173068	-1.001247882
**KIAA0754**	NM_015038	7.55E+10	3.21638417	-1.685439735
**UBR4**	ENST00000375218	0.00025	2.30601835	-1.205403993
**HUWE1**	NM_031407	0.001036	2.54597998	-1.348221075
**LOC100131541**	AY358248	0.000259	4.09708786	-2.034598831
**RUFY2**	NM_001042417	0.000855	2.95314741	-1.562253372
**PHACTR1**	ENST00000379350	0.000835	2.7942214	-1.482446337
**DIDO1**	NM_080797	2.90E+11	2.21032119	-1.144256028
**AP5B1**	NM_138368	0.000762	2.15955639	-1.110734988
**SNX29**	BC029857	0.001585	2.82935047	-1.500470894
**ACVR2B**	NM_001106	0.000774	2.13936472	-1.097182455
**BMS1P5**	NR_003611	0.000931	2.01643753	-1.011808711
**SPDYE5**	NM_001099435	0.000811	2.02685356	-1.019241858
**E2F7**	NM_203394	0.000304	2.37126875	-1.245659181
**SLC30A4**	NM_013309	0.003707	2.12850642	-1.089841442
**PLGLB1**	NM_001032392	0.000633	2.11657405	-1.081730963
**LINC01000**	NR_024368	6.46E+11	2.03459978	-1.024745034
**IRF2BPL**	NM_024496	0.001498	2.0574522	-1.040858913
**HIVEP3**	NM_024503	0.000186	2.16847992	-1.116684084
**PAG1**	NM_018440	0.000411	2.19593239	-1.134833636
**NBPF10**	NM_001039703	0.000329	2.0280571	-1.020098272
**LOC728061**	AK025151	0.004528	2.2925241	-1.1969369
**IBA57**	NM_001010867	0.001085	2.2745564	-1.185585208
**PAG1**	NM_018440	8.53E+09	3.44843411	-1.785941401
**THC2591311**	THC2591311	0.006165	2.07261372	-1.051451262
**A_33_P3220994**	N/A	0.002091	6.00716686	-2.586684738
**LOC101927270**	XR_432672	0.004032	2.32764244	-1.218869456
**LPP**	NM_005578	0.003646	2.40796924	-1.267816963
**OFD1**	NM_003611	7.09E+11	2.07574224	-1.053627305
**A_33_P3225983**	N/A	2.99E+11	2.21899414	-1.149905858
**CD7**	NM_006137	0.000498	2.00708461	-1.005101436
**SLC25A34**	NM_207348	0.000816	2.32178283	-1.215233035
**TDRD6**	NM_001010870	8.50E+10	2.13658547	-1.09530703
**MBTD1**	NM_017643	0.000317	2.29673624	-1.199585185
**GOLGA6L9**	NM_198181	0.008294	2.11158109	-1.078323651
**NF1**	NM_000267	0.006159	2.14273286	-1.099451997
**SLMAP**	ENST00000467901	0.002237	2.12118769	-1.084872281
**FAM71F2**	NM_001290254	0.0007	2.30533075	-1.204973752
**C2CD2**	AK129520	0.001895	3.80056643	-1.926214451

**MAP3K8**	ENST00000375322	0.008473	2.27255154	-1.184313014
**A_33_P3247678**	N/A	0.003823	2.289891	-1.195278927
**A_33_P3255677**	N/A	0.001873	2.69003177	-1.427623211
**ENST00000362058**	ENST00000362058	0.00167	2.17138314	-1.118614312
**CNTRL**	ENST00000373851	0.000105	3.62975621	-1.859872654
**SERP2**	ENST00000474333	0.000335	2.37016487	-1.244987417
**ENST00000501122**	ENST00000501122	0.001848	3.39659214	-1.764087991
**ENST00000413484**	ENST00000413484	0.001824	2.64272356	-1.402025521
**LRRC66**	NM_001024611	2.30E+10	2.62119579	-1.39022512
**THC2524986**	THC2524986	0.001182	2.09768033	-1.068794839
**GREB1L**	NM_001142966	0.000865	2.39037967	-1.257239783
**A_33_P3275163**	N/A	0.005939	2.9778266	-1.574259748
**ENST00000413484**	ENST00000413484	0.004556	2.15155911	-1.105382476
**LINC01000**	NR_024368	0.000112	2.46771288	-1.303174546
**A_33_P3280502**	N/A	0.000701	2.37380552	-1.247201743
**ENST00000479369**	ENST00000479369	0.003569	2.13835478	-1.096501234
**IL6R**	NM_000565	0.000403	3.40214157	-1.766443176
**COX11**	ENST00000572088	0.000106	2.94779086	-1.559634172
**AAK1**	NM_014911	5.84E+11	2.15656686	-1.108736445
**CYP1B1**	NM_000104	0.001384	3.04727221	-1.60751838
**lnc-GAPDH-3**	lnc-GAPDH-3:2	0.007685	2.86324286	-1.517650045
**A_33_P3298980**	N/A	0.000727	2.02469373	-1.017703692
**FAM160B1**	ENST00000369246	0.000298	2.45033383	-1.292978313
**MAN1B1**	ENST00000536349	0.007928	2.59901404	-1.377964427
**PARP4**	NM_006437	0.000253	2.78609753	-1.478245762
**RPS6KA5**	ENST00000614987	6.08E+10	3.11494994	-1.639208978
**ZNF507**	ENST00000587084	0.000413	2.40289903	-1.264776028
**LINC01530**	NR_034159	0.00994	2.18186927	-1.125564663
**PROSER2**	NM_153256	0.003125	2.02263665	-1.016237175
**SH3BP2**	NM_001145855	0.000928	2.38270521	-1.252600471
**LOC100130419**	AK126781	0.006047	2.0207026	-1.014857006
**ZNF345**	NM_003419	0.000376	2.18423462	-1.127127832
**LOC100506191**	AF289610	0.000239	2.12926841	-1.090357824
**ATRX**	NM_000489	0.001162	2.07207704	-1.051077644
**BQ934349**	BQ934349	0.002371	3.54020691	-1.823833682
**ENST00000615493**	ENST00000615493	0.000116	2.57051635	-1.362058189
**ARL4C**	NM_001282431	0.008863	2.00892639	-1.006424703
**STARD9**	ENST00000564158	0.001977	2.11518884	-1.08078647

**LOC100130463**	AK124300	0.001838	2.5452354	-1.347799092
**A_33_P3334121**	N/A	4.16E+09	2.9703629	-1.570639201
**ANKRD36**	NM_001164315	1.90E+11	2.44074607	-1.287322208
**ENST00000424493**	ENST00000424493	0.000129	2.16586709	-1.114944714
**ENST00000479369**	ENST00000479369	0.002676	2.1030972	-1.072515529
**ZNF480**	NM_144684	0.00384	2.02456117	-1.017609233
**SNX29**	BC029857	0.006084	2.1325314	-1.092566984
**UBN2**	NM_173569	6.46E+11	2.10754228	-1.075561574
**GPAM**	ENST00000369425	0.009722	2.04457808	-1.031803159
**PPTC7**	NM_139283	0.000816	2.09126496	-1.064375861
**lnc-CEMP1-1**	lnc-CEMP1-1:2	0.001924	2.6509223	-1.406494385
**LOC101929115**	XR_246488	0.00636	2.07574582	-1.053629793
**A_33_P3351524**	N/A	0.000777	2.56704807	-1.360110312
**GAS6-AS1**	AK092862	0.001195	2.20787859	-1.142660841
**TTC9C**	ENST00000294161	7.46E+10	2.78477788	-1.47756226
**FLJ45482**	AK127393	0.000383	3.09512901	-1.629999545
**DENND2C**	NM_001256404	0.000667	3.11176372	-1.637732519
**RPS15AP10**	NR_026768	0.0002	3.20462966	-1.680157643
**POGZ**	ENST00000485040	0.000328	3.10691977	-1.635484987
**A_33_P3358851**	N/A	0.001211	2.21057844	-1.144423928
**A_33_P3359869**	N/A	0.000147	3.00349164	-1.586640648
**A_33_P3362601**	N/A	0.000192	3.42218709	-1.774918634
**AW571659**	AW571659	0.00019	3.58901381	-1.843587474
**VAPB**	NM_004738	0.00443	2.02027059	-1.014548537
**NBPF12**	NM_001278141	0.000935	2.03257871	-1.023311221
**ARHGAP23**	NM_001199417	0.002019	2.06454468	-1.045823641
**SZT2**	ENST00000470139	0.000636	2.40900993	-1.26844034
**MKI67**	NM_002417	0.002725	2.2814362	-1.18994231
**HNRNPU-AS1**	NR_026778	0.001191	2.16117501	-1.111815905
**RPRD2**	ENST00000369067	0.000144	2.13067055	-1.091307537
**DMTF1**	NR_024549	0.001745	2.04241896	-1.030278836
**FAM215A**	NR_026770	0.000601	2.41895247	-1.274382422
**C5orf56**	AK025221	0.001358	2.30438709	-1.20438308
**A_33_P3404221**	N/A	2.67E+11	2.86075401	-1.516395449
**FGD4**	NM_139241	0.001158	2.17778039	-1.122858478
**ENST00000405431**	ENST00000405431	0.000785	2.49704647	-1.32022267
**THC2560329**	THC2560329	3.66E+11	2.04745913	-1.033834654
**AKAP11**	NM_016248	0.004092	2.01967621	-1.014124022
**FAM43A**	NM_153690	0.001658	2.34842801	-1.231695369
**ENST000004786**	ENST00000478672	0.002599	2.11921573	-1.083530457

**72**				
**PWARSN**	NR_022011	0.009833	2.085958	-1.06071011
**AK126796**	AK126796	3.01E+11	2.04163623	-1.029725836
**LOC102725171**	XR_432995	0.000751	2.18115807	-1.125094327
**XM_006710179**	XM_006710179	0.00201	2.16026354	-1.111207324
**LOC285178**	AK091571	0.008305	2.04253817	-1.030363039
**LOC284581**	AK094426	0.004792	2.38852954	-1.256122719
**MGC12488**	BC005372	1.31E+11	2.58524823	-1.370302812
**LOC284219**	AK094436	0.004335	2.23474121	-1.160107772
**LARS**	NM_020117	0.000859	2.28395677	-1.191535344
**PRO2852**	XR_158845	0.005598	2.30767536	-1.206440282
**lnc-RP11-298P3.4.1-1**	lnc-RP11-298P3.4.1-1:1	0.000154	2.55148506	-1.351337194
**ENST00000565617**	ENST00000565617	1.78E+11	2.49083066	-1.316626943
**LINC01004**	NR_039981	0.002012	2.05579877	-1.039699055
**C8orf60**	AK022255	0.00565	2.14602637	-1.101667804
**MGC24103**	BC020879	0.000403	2.07136106	-1.050579052
**BC015643**	BC015643	0.001401	2.22087598	-1.151128831
**LOC340335**	AK074459	0.001859	2.05849171	-1.041587639
**LOC100996405**	XM_003846604	0.000296	4.82268286	-2.269835941
**ENST00000448494**	ENST00000448494	3.60E+11	2.04559708	-1.032522006

Lists of differentially regulated genes in HGEps after S. oralis biofilm challenge

Table S5. List of upregulated genes in HGEps after 2 hours *S. oralis* biofilm challenge.

Gene name	Systematic name	p	Fold change	log2 difference
**EDN2**	NM_001956	0.001493	0.316414	1.660116703
**CXCL2**	NM_002089	0.007035	0.474041	1.076916568
**TNF**	NM_000594	0.00174	0.273871	1.868432034
**IL6**	NM_000600	0.003107	0.348669	1.520069903
**PDE4B**	NM_001037341	0.006957	0.474189	1.076466039
**ENST00000390597**	ENST00000390597	0.009541	0.295235	1.760066624
**CXCL8**	NM_000584	0.007087	0.27371	1.869281458
**FRY**	NM_023037	0.008224	0.273557	1.870084855
**CXCL1**	NM_001511	0.003937	0.49058	1.027440573
**PRDM1**	NM_001198	0.008165	0.412511	1.277494805
**ARHGAP8**	NM_001198726	0.002551	0.30301	1.722563179

Table S6. List of downregulated genes in human gingival epithelial cells after 2 hours *S. oralis* biofilm challenge.

Gene name	Systematic name	p	Fold change	log2 difference
**ZNF518A**	NM_014803	0.001126	2.05710673	-1.040616648
**TRMT13**	ENST00000370139	0.009544	2.52427936	-1.335871581
**ZNF772**	NM_001024596	0.002298	2.46420121	-1.301120062
**XRCC5**	ENST00000392133	0.008775	2.077034	-1.054524833

Enriched pathways of upregulated genes in tissue cells after S. oralis biofilm challenge

Table S7. List of all enriched pathways in HGFs and HGEps after 2 hours *S. oralis* biofilm challenge.

	Human gingival fibroblasts		Human gingival epithelial cells	
**Pathway**	**p**	Genes	p	Genes
**African trypanosomiasis**			0.0237	IL6, TNF
**Amoebiasis**			0.0023	IL6, TNF, CXCL8
**Amphetamine addiction**	0.0793	FOS, ARC, JUN		
**Chagas disease (American trypanosomiasis)**	0.0061	FOS, IL6, CCL2, JUN, IL12A	0.0022	IL6, TNF, CXCL8
**Chemokine signaling pathway**	0.0421	CXCL1, CCL2, NCF1, CXCL3, CXCL2	0.0068	CXCL1, CXCL2, CXCL8
**Colorectal cancer**	0.0712	FOS, JUN, MYC		
**Cytokine-cytokine receptor interaction**	0.0791	CSF2, IL6, CCL2, IL12A, TNFSF9	0.0103	IL6, TNF, CXCL8
**FoxO signaling pathway**	0.0003	SGK1, IL6, PLK3, PLK2, GADD45G, GADD45B, KLF2		
**Graft-versus-host disease**			0.0237	IL6, TNF
**Hematopoietic cell lineage**			0.0600	IL6, TNF
**Hepatitis B**	0.0190	FOS, IL6, EGR2, JUN, MYC	0.0042	IL6, TNF, CXCL8
**Hepatitis C**			0.0926	TNF, CXCL8
**Herpes simplex infection**	0.0092	FOS, IL6, CCL2, SOCS3, JUN, IL12A		
**HTLV-I infection**	0.0020	ZFP36, EGR1, CSF2, FOS, IL6, EGR2, JUN, MYC		
**Hypertrophic cardiomyopathy (HCM)**			0.0552	IL6, TNF
**Inflammatory bowel disease (IBD)**	0.0752	IL6, JUN, IL12A	0.0455	IL6, TNF
**Influenza A**	0.0342	IL6, CCL2, SOCS3, JUN, IL12A	0.0060	IL6, TNF, CXCL8
**Insulin resistance**			0.0758	IL6, TNF
**Jak-STAT signaling pathway**	0.0190	CSF2, IL6, SOCS3, IL12A, MYC		
**Legionellosis**	0.0005	CXCL1, IL6, CXCL3, CXCL2, IL12A	0.0000	CXCL1, IL6, TNF, CXCL2, CXCL8
**Leishmaniasis**	0.0137	FOS, NCF1, JUN, IL12A		
**Malaria**	0.0469	IL6, CCL2, IL12A	0.0005	IL6, TNF, CXCL8
**MAPK signaling pathway**	0.0001	DUSP5, FGF5, FOS, DUSP2, DUSP1, JUN, GADD45G, GADD45B, MYC, NGF		
**NF-kappa B signaling pathway**			0.0614	TNF, CXCL8
**NOD-like receptor signaling pathway**			0.0006	IL6, TNF, CXCL8
**Non-alcoholic fatty liver disease (NAFLD)**			0.0045	IL6, TNF, CXCL8
**Osteoclast differentiation**	0.0135	FOS, SOCS3, NCF1, JUN, JUNB		
**p53 signaling pathway**	0.0814	GADD45G, PMAIP1,		

		GADD45B		
**Pertussis**	0.0158	FOS, IL6, JUN, IL12A	0.0011	IL6, TNF, CXCL8
**PI3K-Akt signaling pathway**	0.0959	FGF5, SGK1, IL6, MYC, EPHA2, NGF		
**Rheumatoid arthritis**	0.0034	CSF2, FOS, IL6, CCL2, JUN	0.0016	IL6, TNF, CXCL8
**RIG-I-like receptor signaling pathway**			0.0496	TNF, CXCL8
**Salmonella infection**	0.0000	CXCL1, CSF2, FOS, IL6, CXCL3, JUN, CXCL2	0.0000	CXCL1, IL6, CXCL2, CXCL8
**Systemic lupus erythematosus**	0.0689	HIST2H3A, HIST4H4, HIST1H4A, HIST1H4E		
**TNF signaling pathway**	0.0000	CXCL1, CSF2, FOS, IL6, CCL2, SOCS3, CXCL3, JUN, EDN1, CXCL2, JUNB	0.0000	CXCL1, IL6, TNF, CXCL2
**Toll-like receptor signaling pathway**	0.0389	FOS, IL6, JUN, IL12A	0.0023	IL6, TNF, CXCL8
**Transcriptional misregulation in cancer**	0.0306	HIST2H3A, CSF2, HHEX, IL6, MYC		
**Viral carcinogenesis**	0.0145	HIST4H4, EGR2, HIST1H4A, JUN, HIST1H4E, PMAIP1		
**Wnt signaling pathway**	0.0739	DKK1, JUN, BAMBI, MYC		

Cell nuclei of gingival tissue cells on titanium disks after S. oralis biofilm challenge

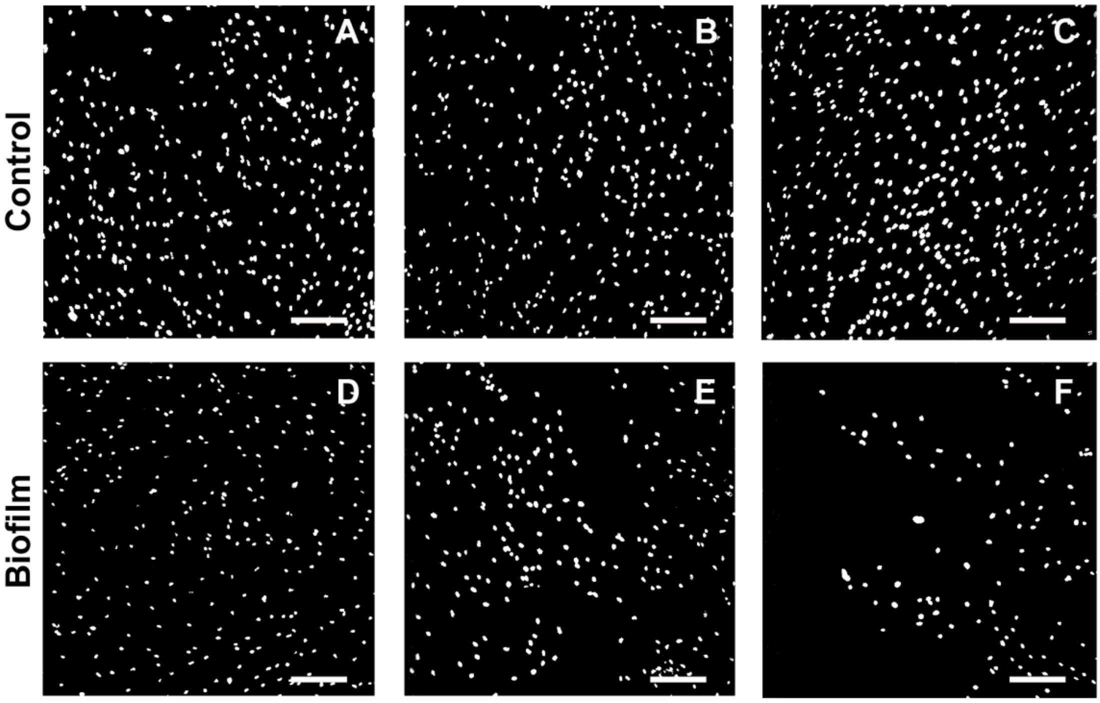

Figure S1. Cell nuclei of HGFs on titanium disks after *S. oralis* biofilm challenge. HGFs grown on titanium disks were co-cultured with the *S. oralis* biofilm or a control membrane for up to 6 h. Subsequently, the cells were fixed after examination of the LIVE/DEAD staining and their nuclei were stained with DAPI (white). The distribution of cell nuclei remained similar in the control samples after 2, 4 or 6 h - Figures (A), (B), and (C), respectively. Also, after 2 h of co-culture with the *S. oralis* biofilm, the distribution of cell nuclei was similar to that of the control (D). Less cell nuclei were detected after 4 h (E) and much less after 6 h (F). Typical pictures from two independent experiments. Scale bars: 200 μm

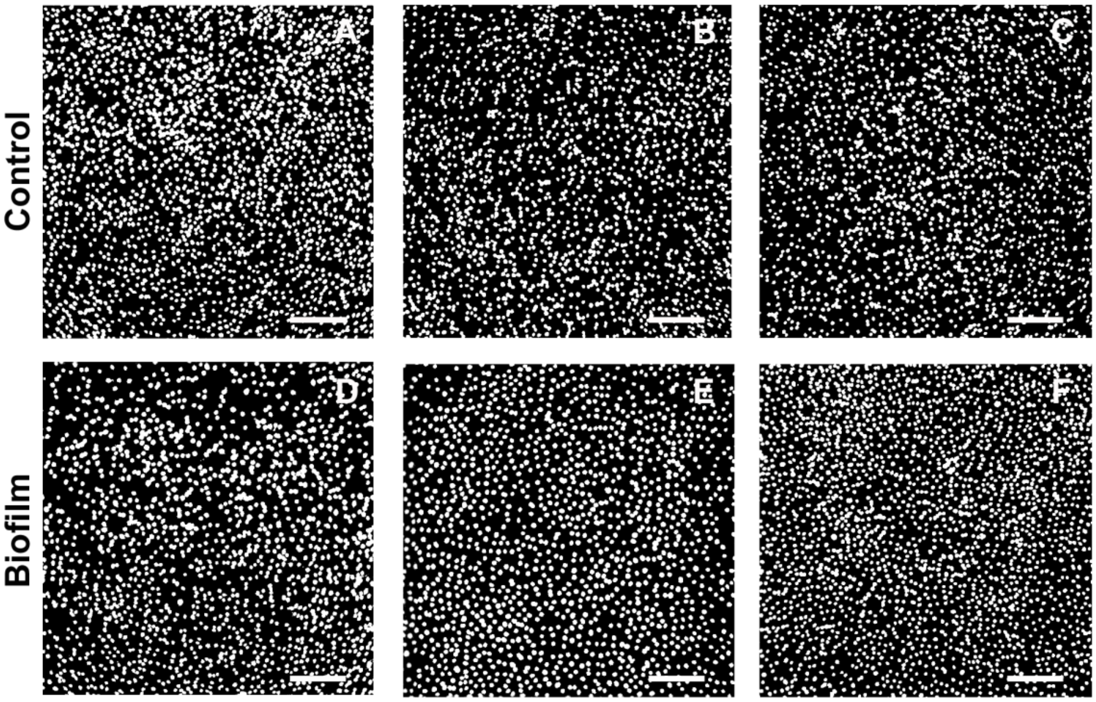

Figure S2. Cell nuclei of HGEps on titanium disks after *S. oralis* biofilm challenge. HGEps grown on titanium disks were co-cultured with the *S. oralis* biofilm or a control membrane for up to 6 h. Subsequently, the cells were fixed after examination of the LIVE/DEAD staining and their nuclei were stained with DAPI (white). The distribution of cell nuclei remained similar in the control samples after 2, 4 or 6 h - Figures (A), (B), and (C), respectively. Also, after co-culture with the *S. oralis* biofilm for 2, 4 or 6 h - Figures (D), (E), and (F), respectively, the distribution of cell nuclei was similar to that of the controls. Typical pictures from two independent experiments. Scale bars: 200 μm

Effect of tissue cell culture medium on S. oralis biofilm

In order to determine the effects of the tissue cell culture media on the volume and vitality of the *S. oralis* biofilm, they were cultured for 2 hours in the same setting as for the co-cultures without tissue cells on titanium disks. After incubation, the biofilms were washed once with PBS and stained with the LIVE/DEAD®BacLight™ Bacterial Viability Kit (Life Technologies, Darmstadt, Germany). Briefly, the biofilms were fluorescently stained with SYTO9 and propidium iodide as a 1:1,000 dilution in PBS for 30 min. Then, they were washed with PBS and fixed for 5 min with 2.5% glutardialdehyde in PBS. The stained biofilms were scanned at three random positions with a 40-fold magnification by CLSM (Leica TCS SP2). The acquired z-plane images were reconstructed into 3D images by the Imaris® x 64 6.2.1 software package (Bitplane). Subsequently, the biofilm volume (μm^3) was calculated applying the surpass mode. The live and dead percentages were calculated after setting the total biofilm volume to 100%.

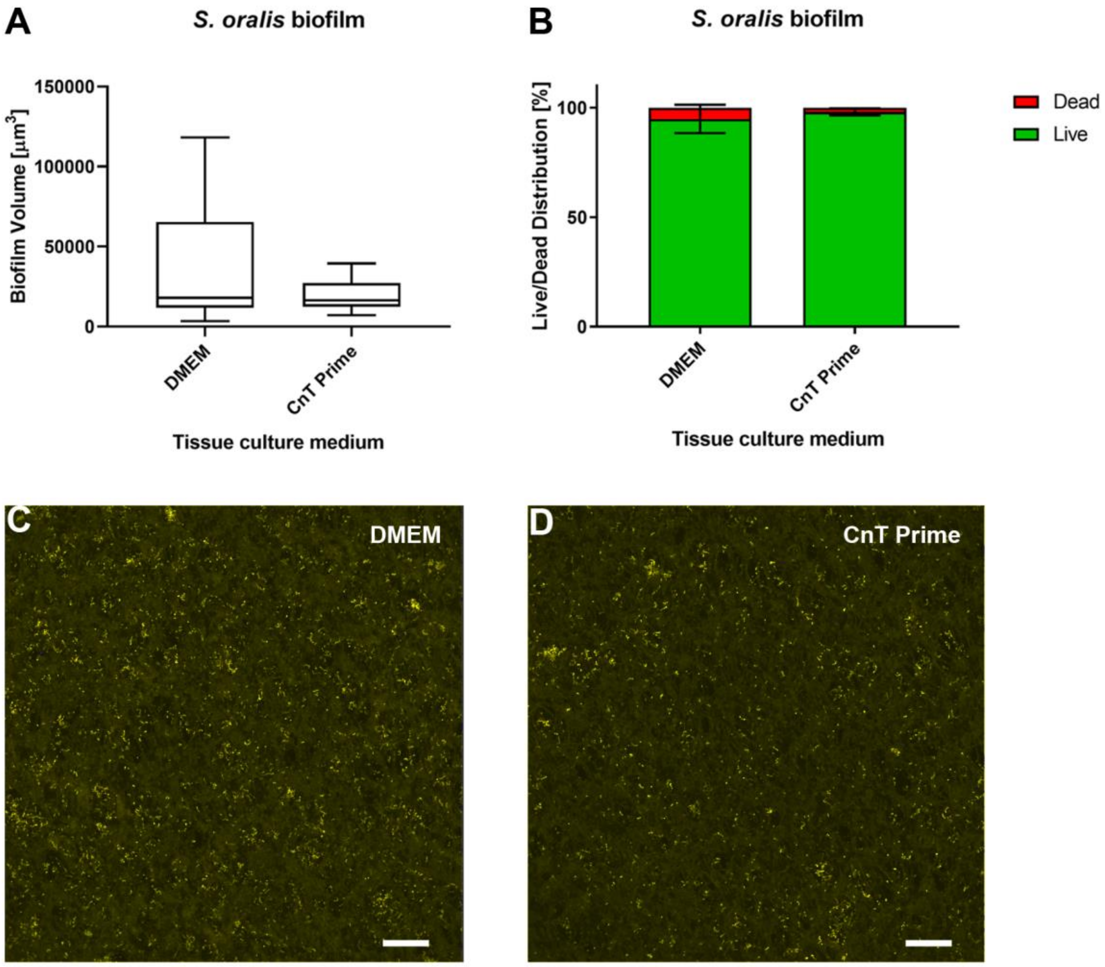

Figure S3. Effect of cell culture media on *S. oralis* biofilm volume and vitality. The biofilms were placed for 2 hours in the DMEM or CnT Prime medium, which were used for the co-cultures with human gingival fibroblasts and epithelial cells, respectively. Bacterial LIVE/DEAD staining was performed to calculate the biofilm volume as well as live and dead proportions of three independent experiments and nine samples in total. The biofilm volume is presented as Box & Whiskers with Tukey error bars (A). Live and dead proportions are depicted in graph bars with error bars indicating the standard deviation (B). Microscopic pictures show *S. oralis* biofilms after culture in DMEM (C) or CnT Prime (D). Live bacteria are depicted in yellow and dead in blue. Error bars: 30 µm.

Role of differentially regulated genes in HGF and HGEp within common enriched pathways

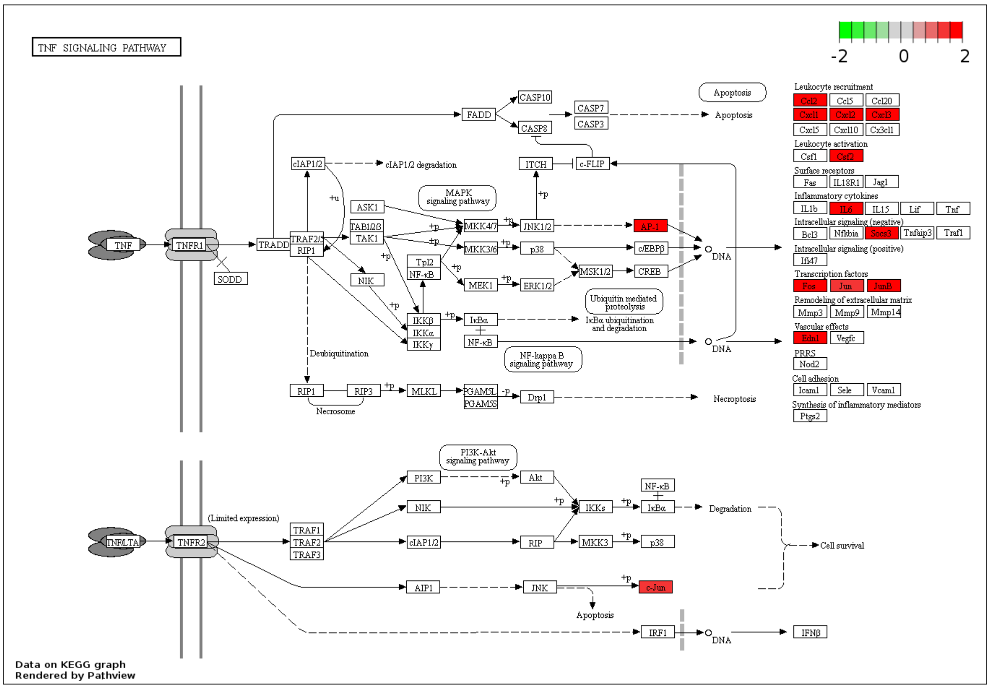

Figure S4. Differentially regulated genes in HGF within the TNF signaling pathway, which was adapted from Pathview using the KEGG (Kyoto Encyclopedia of Genes and Genomes) nomenclature. Red color indicates up-regulation and green indicates downregulation. An arrow indicates a molecular interaction resulting in activation, and a line without an arrowhead indicates a molecular interaction resulting in inhibition. The intensity of the color indicates the difference in expression. +p = phosphorylation, -p = dephosphorylation, +u = ubiquitination, -u = deubiquitination, +m = methylation, e = expression.

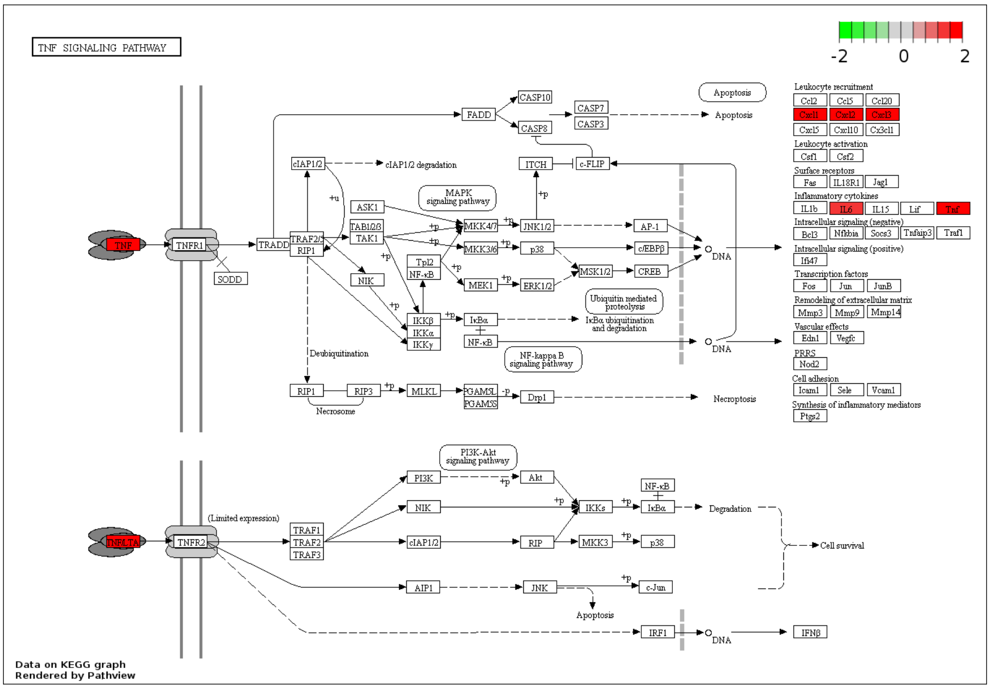

Figure S5. Differentially regulated genes in HGEp within the TNF signaling pathway, which was adapted from Pathview using the KEGG (Kyoto Encyclopedia of Genes and Genomes) nomenclature. Red color indicates up-regulation and green indicates downregulation. An arrow indicates a molecular interaction resulting in activation, and a line without an arrowhead indicates a

molecular interaction resulting in inhibition. The intensity of the color indicates the difference in expression. +p = phosphorylation, -p = dephosphorylation, +u = ubiquitination, -u = deubiquitination, +m = methylation, e = expression.

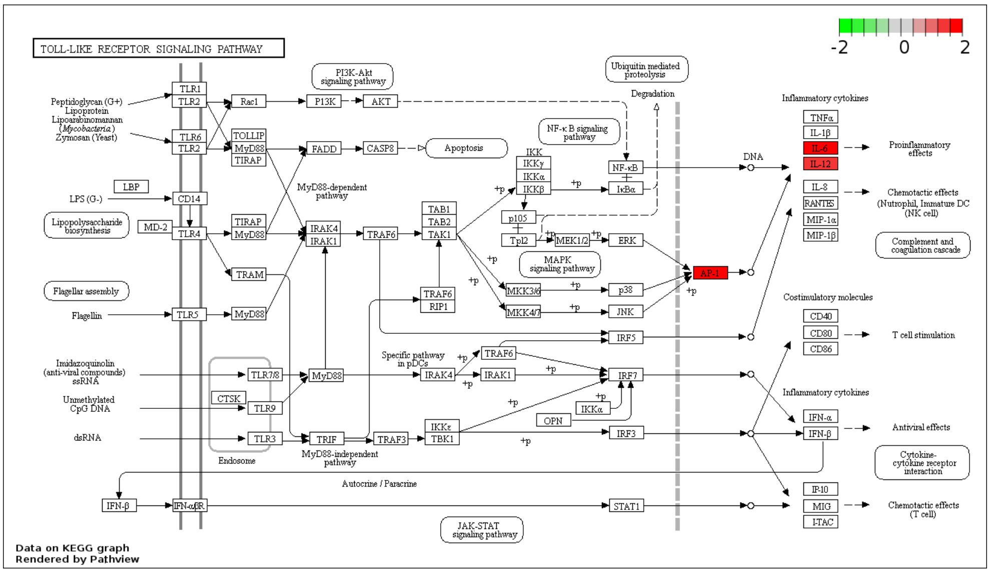

Figure S6. Differentially regulated genes in HGF within the toll-like receptor signaling pathway, which was adapted from Pathview using the KEGG (Kyoto Encyclopedia of Genes and Genomes) nomenclature. Red color indicates up-regulation and green indicates downregulation. An arrow indicates a molecular interaction resulting in activation, and a line without an arrowhead indicates a molecular interaction resulting in inhibition. The intensity of the color indicates the difference in expression. +p = phosphorylation, -p = dephosphorylation, +u = ubiquitination, -u = deubiquitination, +m = methylation, e = expression.

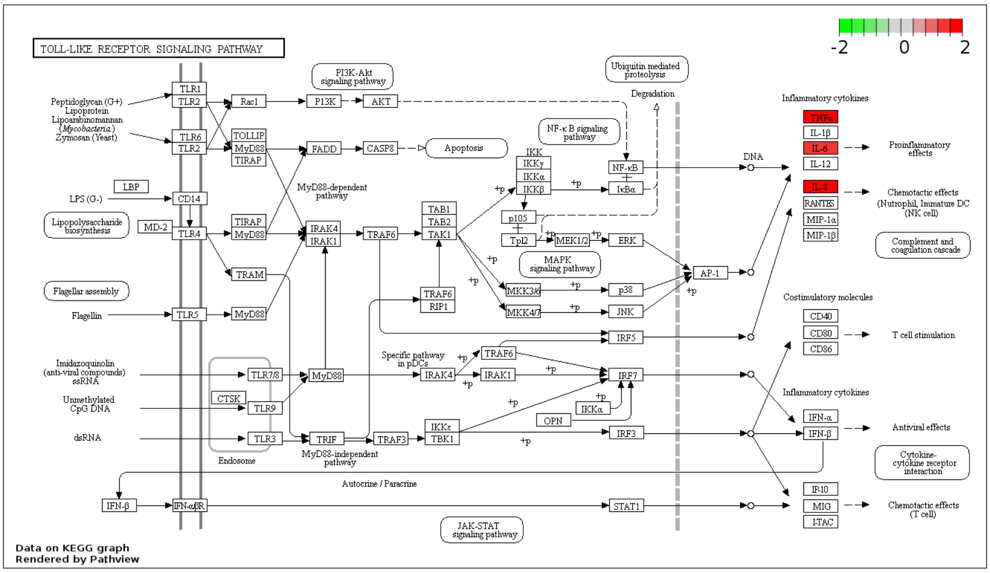

Figure S7. Differentially regulated genes in HGEp within the toll-like receptor signaling pathway, which was adapted from Pathview using the KEGG (Kyoto Encyclopedia of Genes and Genomes) nomenclature. Red color indicates up-regulation and green indicates downregulation. An arrow indicates a molecular interaction resulting in activation, and a line without an arrowhead indicates a molecular interaction resulting in inhibition. The intensity of the color indicates the difference in expression. +p = phosphorylation, -p = dephosphorylation, +u = ubiquitination, -u = deubiquitination, +m = methylation, e = expression.

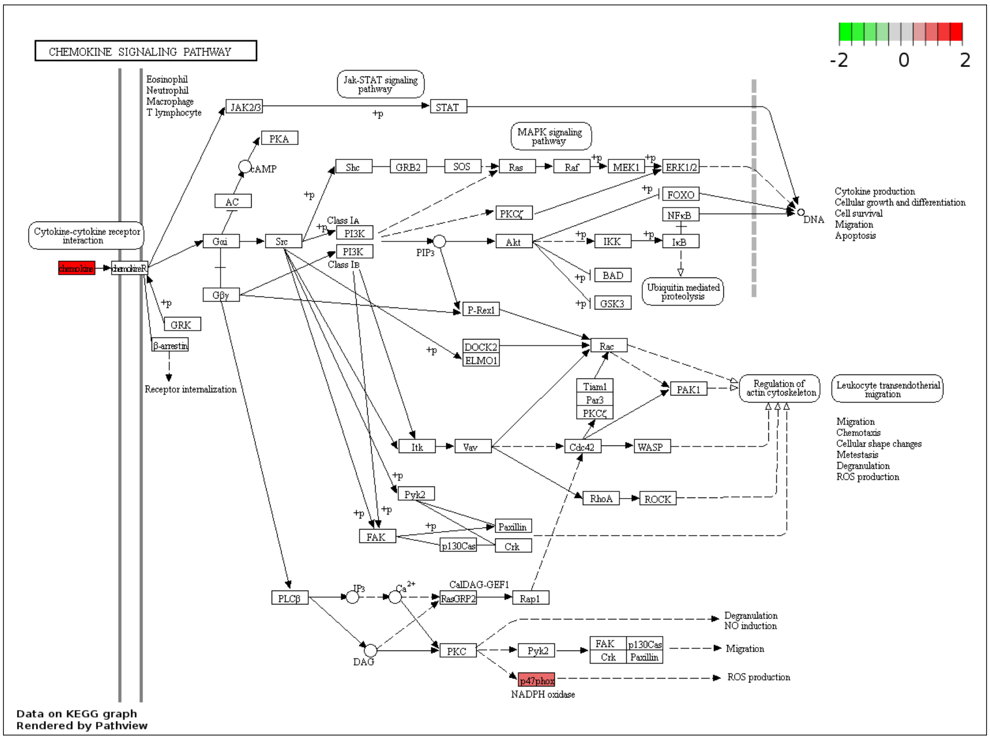

Figure S8. Differentially regulated genes in HGF within the chemokine signaling pathway, which was adapted from Pathview using the KEGG (Kyoto Encyclopedia of Genes and Genomes) nomenclature. Red color indicates up-regulation and green indicates downregulation. An arrow indicates a molecular interaction resulting in activation, and a line without an arrowhead indicates a molecular interaction resulting in inhibition. The intensity of the color indicates the difference in expression. +p = phosphorylation, -p = dephosphorylation, +u = ubiquitination, -u = deubiquitination, +m = methylation, e = expression.

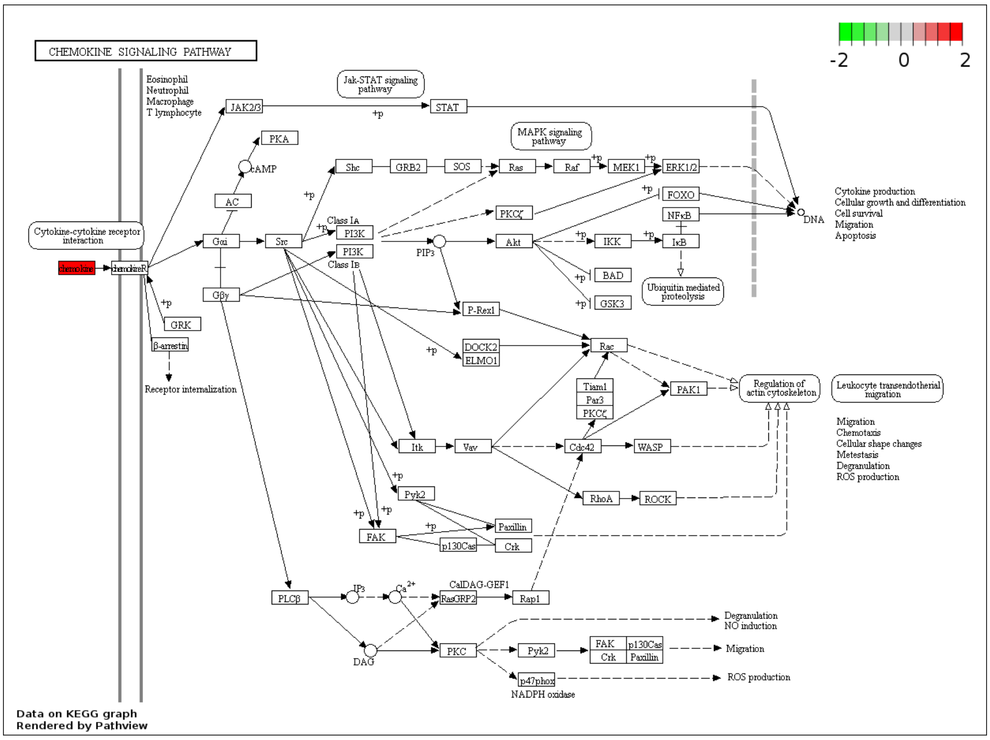

Figure S9. Differentially regulated genes in HGEp within the chemokine signaling pathway, which was adapted from Pathview using the KEGG (Kyoto Encyclopedia of Genes and Genomes) nomenclature. Red color indicates up-regulation and green indicates downregulation. An arrow

indicates a molecular interaction resulting in activation, and a line without an arrowhead indicates a molecular interaction resulting in inhibition. The intensity of the color indicates the difference in expression. +p = phosphorylation, -p = dephosphorylation, +u = ubiquitination, -u = deubiquitination, +m = methylation, e = expression.

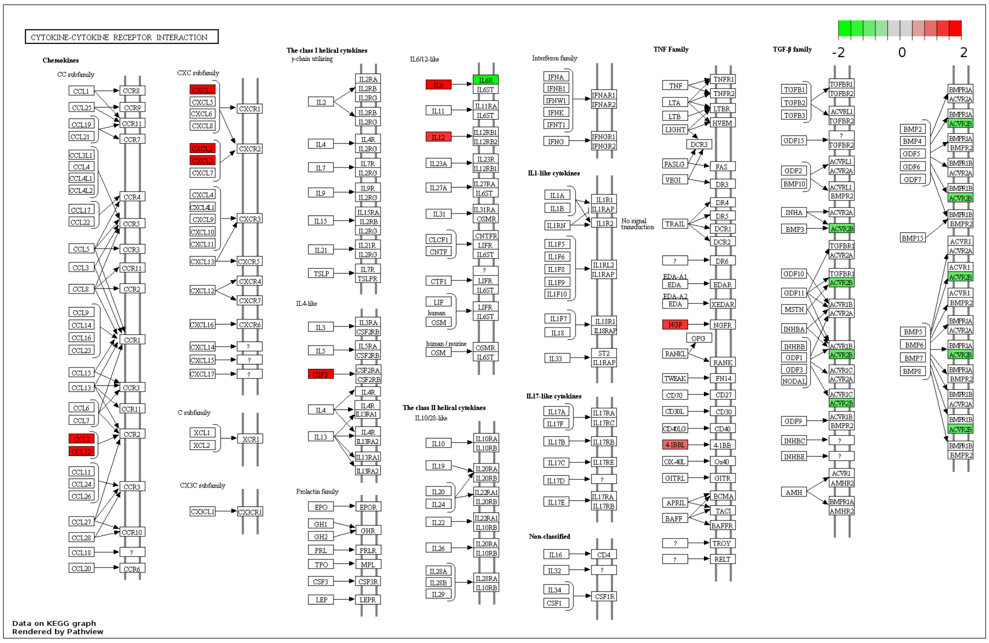

Figure S10. Differentially regulated genes in HGF within the cytokine-cytokine receptor interaction, which was adapted from Pathview using the KEGG (Kyoto Encyclopedia of Genes and Genomes) nomenclature. Red color indicates up-regulation and green indicates downregulation. An arrow indicates a molecular interaction resulting in activation, and a line without an arrowhead indicates a molecular interaction resulting in inhibition. The intensity of the color indicates the difference in expression. +p = phosphorylation, -p = dephosphorylation, +u = ubiquitination, -u = deubiquitination, +m = methylation, e = expression.

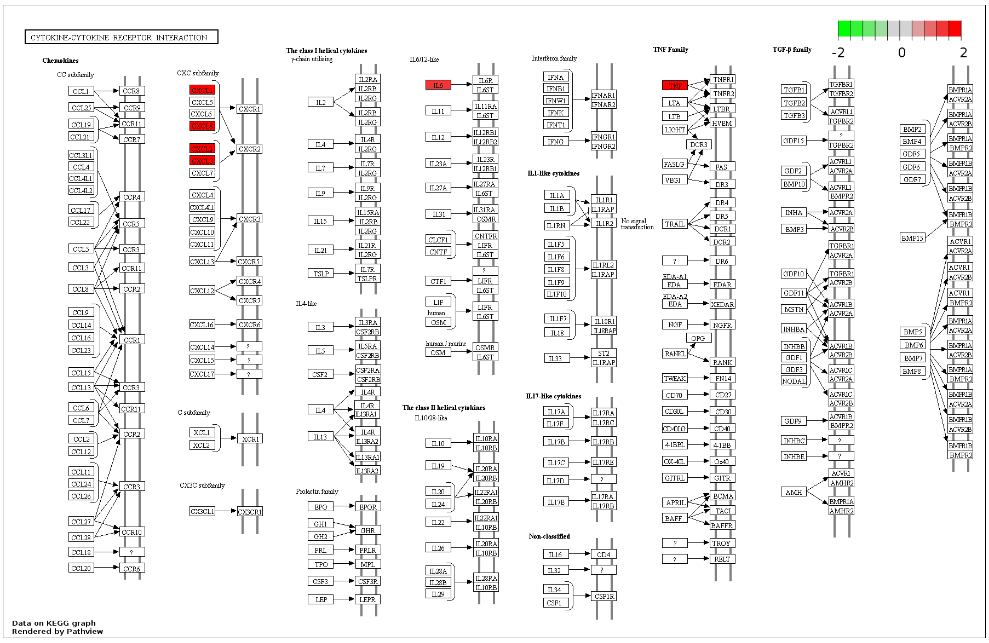

Figure S11. Differentially regulated genes in HGEp within the cytokine-cytokine receptor interaction, which was adapted from Pathview using the KEGG (Kyoto Encyclopedia of Genes and Genomes) nomenclature. Red color indicates up-regulation and green indicates downregulation. An

arrow indicates a molecular interaction resulting in activation, and a line without an arrowhead indicates a molecular interaction resulting in inhibition. The intensity of the color indicates the difference in expression. +p = phosphorylation, -p = dephosphorylation, +u = ubiquitination, -u = deubiquitination, +m = methylation, e = expression.

Role of differentially regulated genes in HGF within the enriched pathways

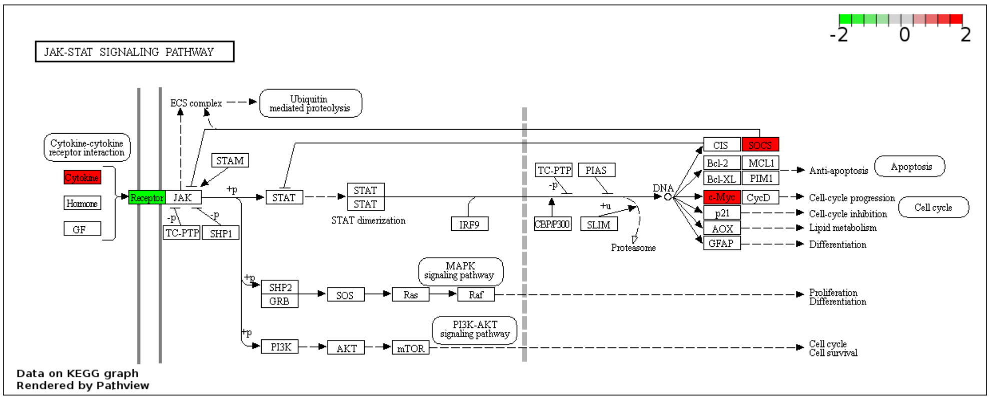

Figure S12. Differentially regulated genes in HGF within the JAK-STAT signaling pathway, which was adapted from Pathview using the KEGG (Kyoto Encyclopedia of Genes and Genomes) nomenclature. Red color indicates up-regulation and green indicates downregulation. An arrow indicates a molecular interaction resulting in activation, and a line without an arrowhead indicates a molecular interaction resulting in inhibition. The intensity of the color indicates the difference in expression. +p = phosphorylation, -p = dephosphorylation, +u = ubiquitination, -u = deubiquitination, +m = methylation, e = expression.

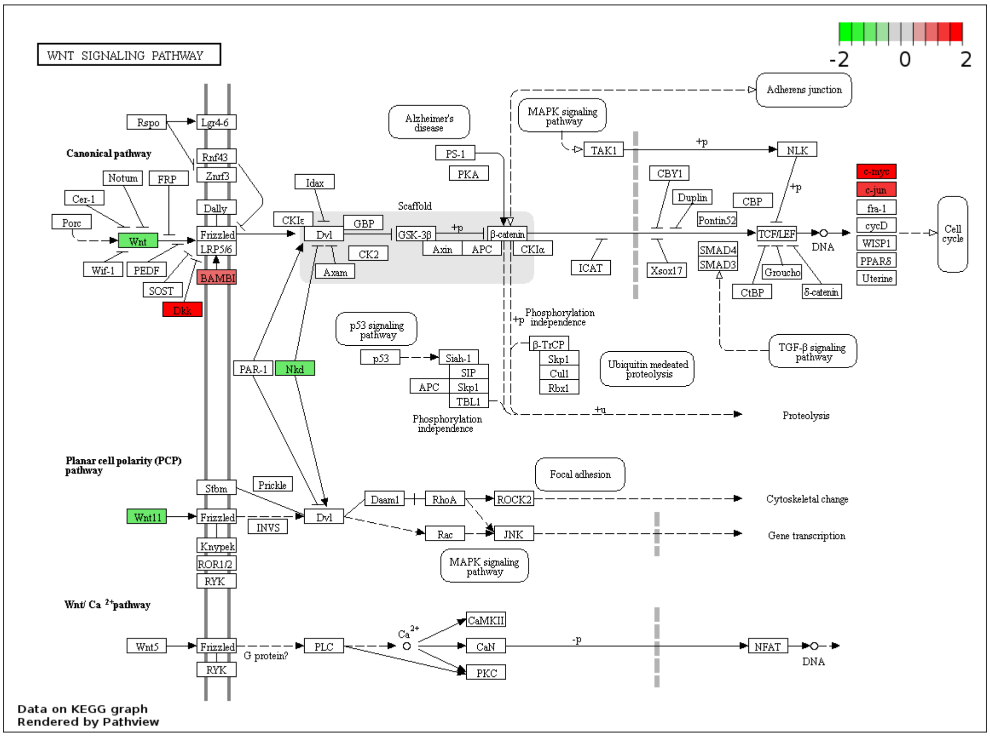

Figure S13. Differentially regulated genes in HGF within the Wnt signaling pathway, which was adapted from Pathview using the KEGG (Kyoto Encyclopedia of Genes and Genomes) nomenclature. Red color indicates up-regulation and green indicates downregulation. An arrow indicates a molecular interaction resulting in activation, and a line without an arrowhead indicates a molecular interaction resulting in inhibition. The intensity of the color indicates the difference in expression. +p = phosphorylation, -p = dephosphorylation, +u = ubiquitination, -u = deubiquitination, +m = methylation, e = expression.

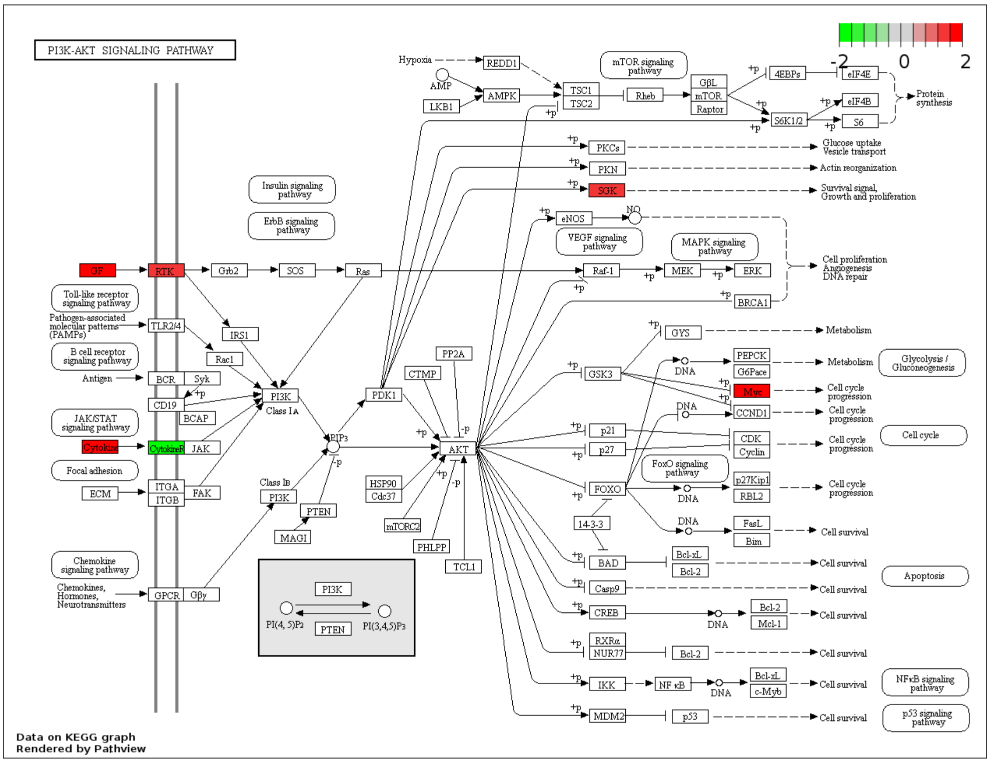

Figure S14. Differentially regulated genes in HGF within the PI3K-Akt signaling pathway, which was adapted from Pathview using the KEGG (Kyoto Encyclopedia of Genes and Genomes) nomenclature. Red color indicates up-regulation and green indicates downregulation. An arrow indicates a molecular interaction resulting in activation, and a line without an arrowhead indicates a molecular interaction resulting in inhibition. The intensity of the color indicates the difference in expression. +p = phosphorylation, -p = dephosphorylation, +u = ubiquitination, -u = deubiquitination, +m = methylation, e = expression.

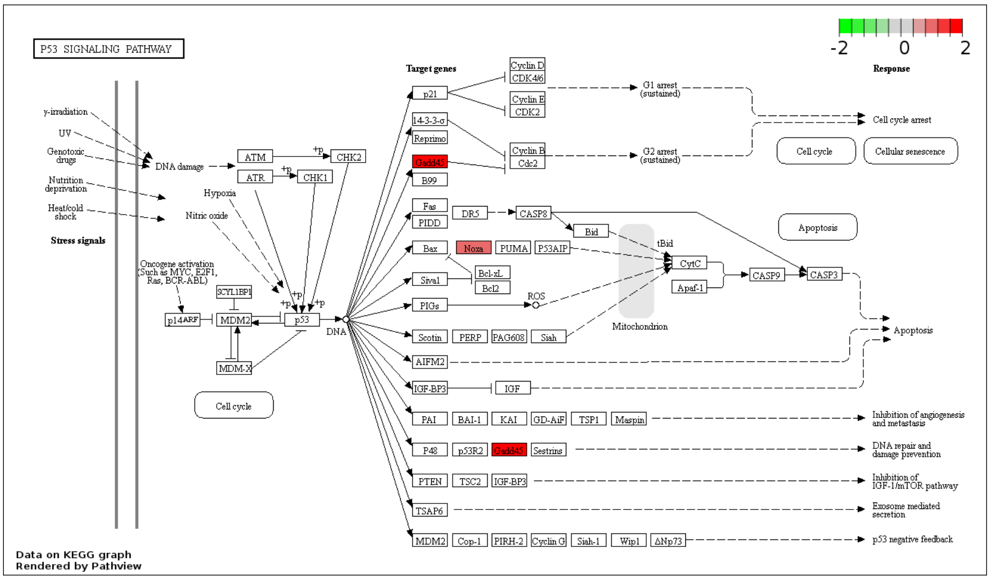

Figure S15. Differentially regulated genes in HGF within the p53 signaling pathway, which was adapted from Pathview using the KEGG (Kyoto Encyclopedia of Genes and Genomes) nomenclature. Red color indicates up-regulation and green indicates downregulation. An arrow indicates a molecular interaction resulting in activation, and a line without an arrowhead indicates a molecular interaction resulting in inhibition. The intensity of the color indicates the difference in

expression. +p = phosphorylation, -p = dephosphorylation, +u = ubiquitination, -u = deubiquitination, +m = methylation, e = expression.

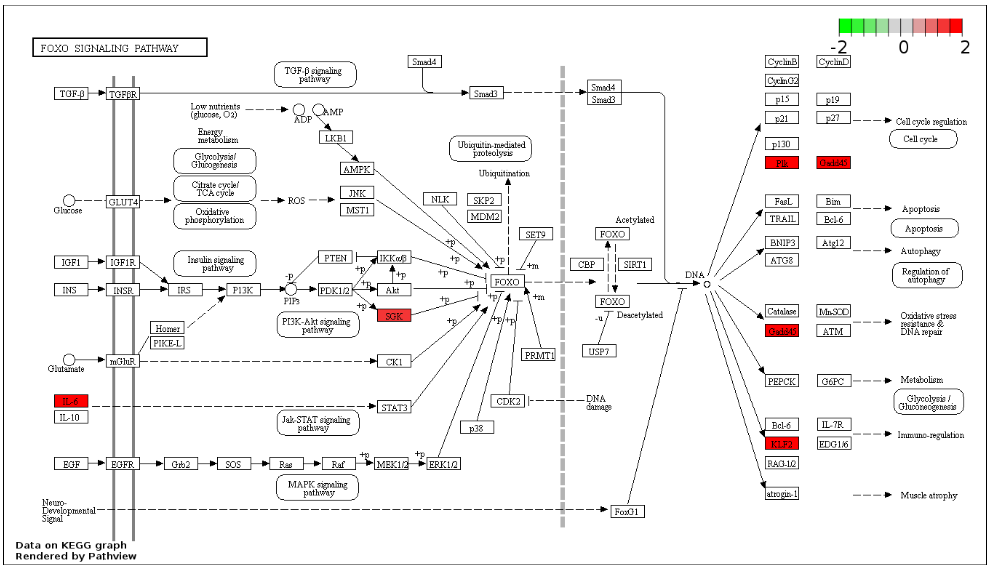

Figure S16. Differentially regulated genes in HGF within the FoxO signaling pathway, which was adapted from Pathview using the KEGG (Kyoto Encyclopedia of Genes and Genomes) nomenclature. Red color indicates up-regulation and green indicates downregulation. An arrow indicates a molecular interaction resulting in activation, and a line without an arrowhead indicates a molecular interaction resulting in inhibition. The intensity of the color indicates the difference in expression. +p = phosphorylation, -p = dephosphorylation, +u = ubiquitination, -u = deubiquitination, +m = methylation, e = expression.

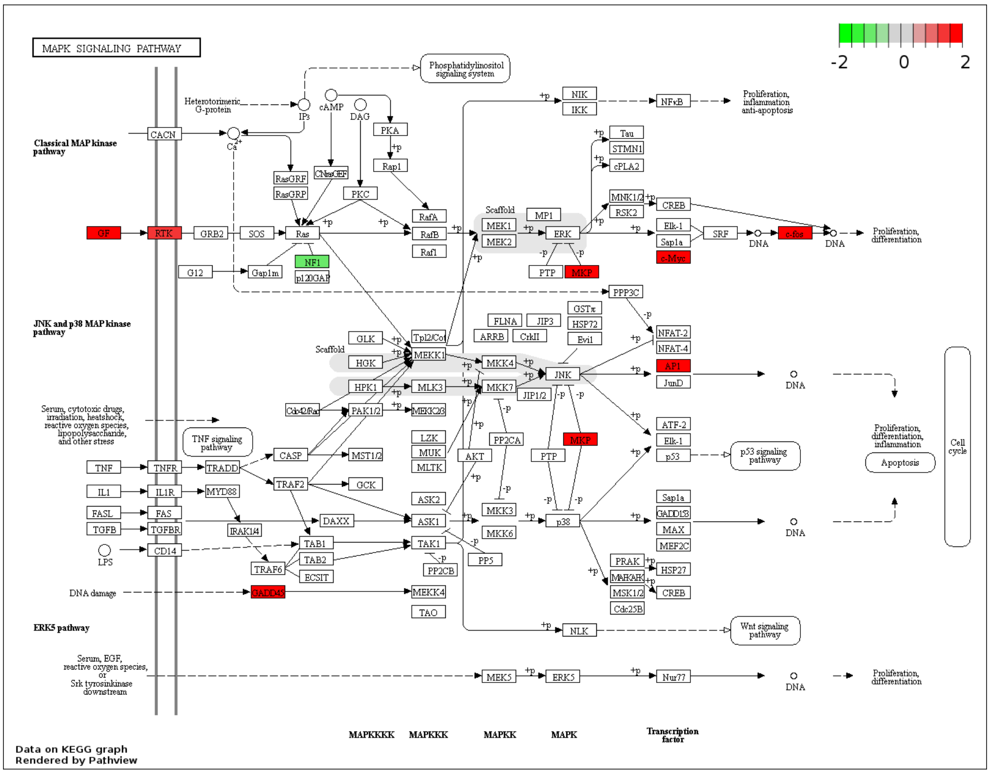

Figure S17. Differentially regulated genes in HGF within the MAPK signaling pathway, which was adapted from Pathview using the KEGG (Kyoto Encyclopedia of Genes and Genomes) nomenclature. Red color indicates up-regulation and green indicates downregulation. An arrow indicates a molecular interaction resulting in activation, and a line without an arrowhead indicates a

molecular interaction resulting in inhibition. The intensity of the color indicates the difference in expression. +p = phosphorylation, -p = dephosphorylation, +u = ubiquitination, -u = deubiquitination, +m = methylation, e = expression.

Role of differentially regulated genes in HGEp within the enriched pathways

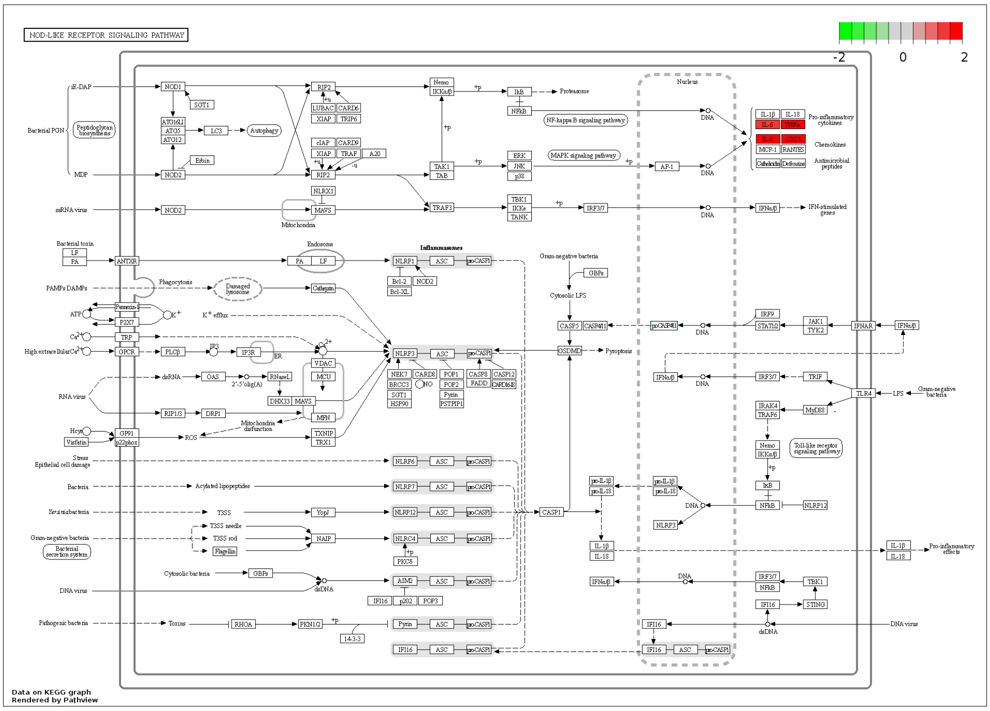

Figure S18. Differentially regulated genes in HGEp within the NOD-like receptor signaling pathway, which was adapted from Pathview using the KEGG (Kyoto Encyclopedia of Genes and Genomes) nomenclature. Red color indicates up-regulation and green indicates downregulation. An arrow indicates a molecular interaction resulting in activation, and a line without an arrowhead indicates a molecular interaction resulting in inhibition. The intensity of the color indicates the difference in expression. +p = phosphorylation, -p = dephosphorylation, +u = ubiquitination, -u = deubiquitination, +m = methylation, e = expression.

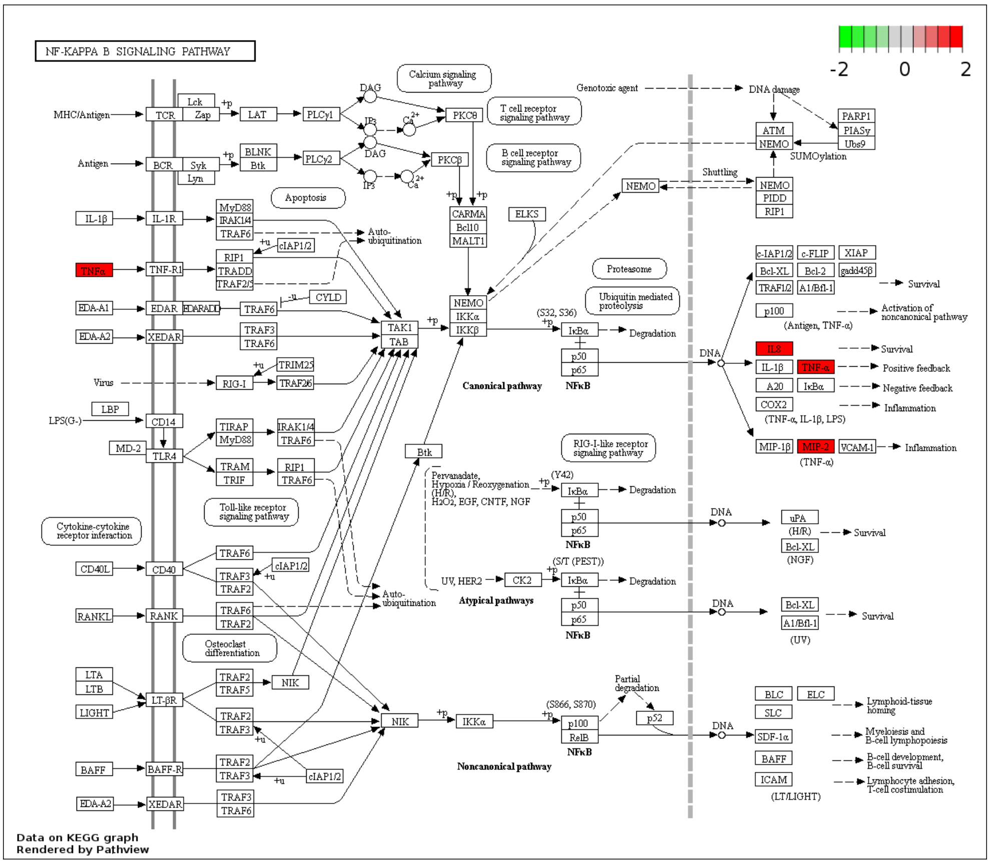

Figure S19. Differentially regulated genes in HGEp within the NF-kappa B signaling pathway, which was adapted from Pathview using the KEGG (Kyoto Encyclopedia of Genes and Genomes) nomenclature. Red color indicates up-regulation and green indicates downregulation. An arrow indicates a molecular interaction resulting in activation, and a line without an arrowhead indicates a molecular interaction resulting in inhibition. The intensity of the color indicates the difference in expression. +p = phosphorylation, -p = dephosphorylation, +u = ubiquitination, -u = deubiquitination, +m = methylation, e = expression.

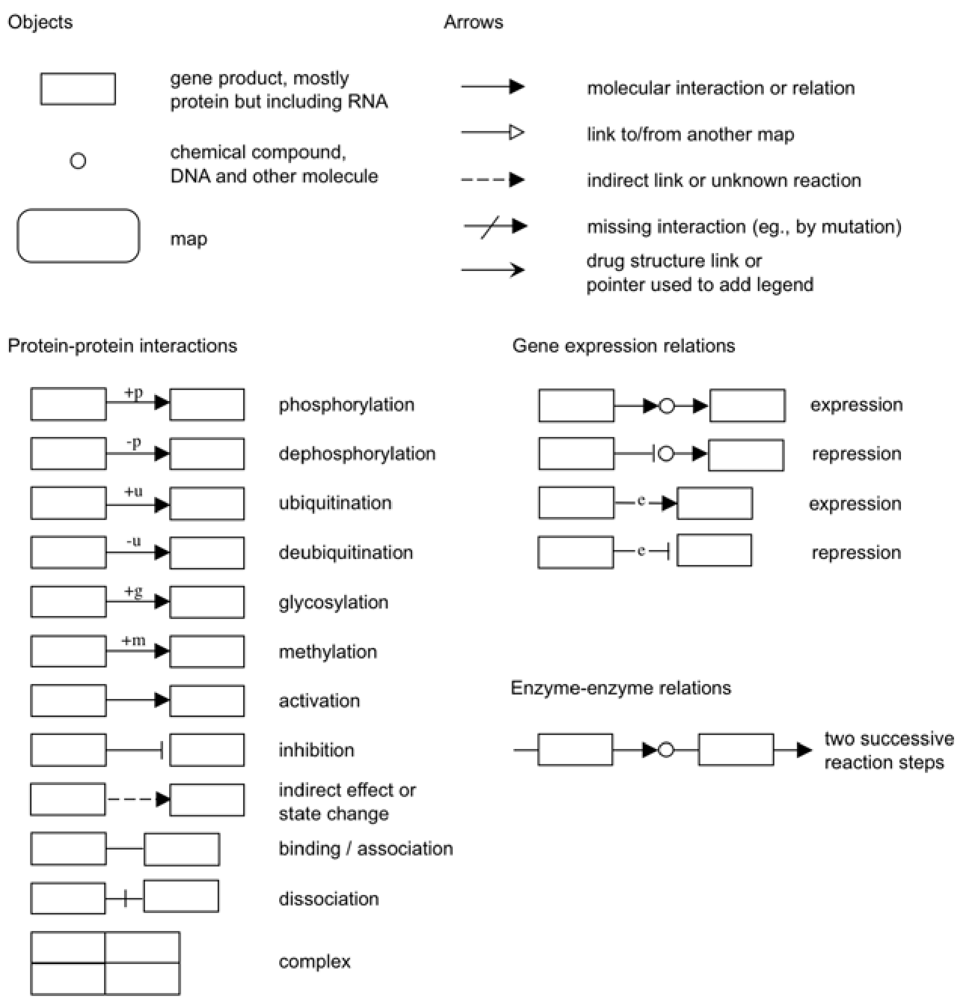

Figure S20. KEGG notation for pathways https://www.genome.jp/kegg/document/help_pathway.html.
